# Effectiveness of respiratory muscle training in adults with multiple sclerosis: a systematic review and meta-analysis

**DOI:** 10.3389/fneur.2025.1665651

**Published:** 2025-10-23

**Authors:** Yuping Xiang, Oufeng Tang, Ling Zeng

**Affiliations:** ^1^Department of Critical Care Medicine, West China Hospital, West China School of Nursing, Sichuan University, Chengdu, Sichuan, China; ^2^Department of Anesthesiology, West China Hospital, West China School of Nursing, Sichuan University, Chengdu, Sichuan, China

**Keywords:** multiple sclerosis, respiratory muscle training, maximum inspiratory pressure, maximum expiratory pressure, meta-analysis, systematic review

## Abstract

**Aims:**

To systematically evaluate the effects of respiratory muscle training (RMT) on respiratory muscle strength, lung function, fatigue, and quality of life in patients with multiple sclerosis (MS).

**Methods:**

Four electronic bibliographic databases (PubMed, Web of Science, Embase, and Cochrane) were searched from inception to August 26, 2024. The screened trials compared RMT with sham RMT as well as conventional care. Two authors independently extracted key information from the eligible studies. A risk of bias assessment was conducted for randomized controlled trials (RCTs) and quasi-experimental (QE) studies using the RoB 2.0 and JBI critical appraisal tools. We assessed the certainty of the evidence according to the GRADE approach applied to the primary outcomes of respiratory muscle strength. Where feasible, the data were pooled and subjected to meta-analysis using RevMan 5.4 software. The results are reported as mean differences (MDs) and 95% confidence intervals (CIs).

**Results:**

A total of 14 trials (eight RCTs and six QE studies) involving 376 patients were included in the analysis. For the primary outcomes, RMT demonstrated significant improvements in maximum inspiratory pressure (MIP) (MD 4.74 cmH_2_O, 95%CI 0.48–9.01, *p* = 0.03), predicted MIP (MD 14.27, 95%CI 2.45–26.09, *p* = 0.02), and maximum expiratory pressure (MEP) (MD 8.50 cmH_2_O, 95%CI 1.59–15.42, *p* = 0.02); however, no statistically significant effect was observed for predicted MEP (MD 2.25, 95%CI -2.36–6.86, *p* = 0.34). For secondary outcomes, RMT failed to show a significant summary effect size on lung function and exercise capacity; however, it showed significantly reduced fatigue (MD −15.15, 95%CI -21.14– −9.16, *p* < 0.00001), as assessed using a modified fatigue impact scale. Due to the limited number of studies, qualitative analysis was used to assess quality of life (QOL), adherence to treatment, and adverse events.

**Conclusion:**

Respiratory muscle training improves respiratory muscle strength and fatigue in MS, but evidence quality is low and effects on lung function, exercise capacity and QOL remain uncertain. The evidence was limited by the small number of trials with small sample sizes and the risk of bias. This necessitates additional randomized controlled trials.

**Systematic review registration:**

https://www.crd.york.ac.uk/prospero/, identifier CRD42023457664.

## 1 Introduction

The global prevalence of multiple sclerosis (MS) has increased; the 2020 estimated number of patients with MS worldwide was 2.8 million (1). MS is one of the most common causes of neurological disability in young adults (2), posing a significant burden on the affected patients and health care systems. For instance, in the United States, the estimated annual total economic burden due to MS is $85.4 billion (3), with an all-cause standardized mortality ratio of 2.61 (4). MS is a complex, autoimmune-mediated disease of the central nervous system characterized by inflammatory demyelination and axonal/neuronal damage that interferes with motor pathways, leading to muscle weakness, especially in the respiratory muscles (5–7). Poor ventilation and coughing ensue from respiratory muscle weakness, which may lead to aspiration, pneumonia, or even acute ventilation failure (8, 9). This can also increase the mortality associated with advanced MS (10), which correlates with the MS-induced disability level (11). Respiratory muscle weakness is significantly associated with decreased cardiorespiratory fitness, airway clearance disorder, speech disorder, dysphagia, urinary incontinence, sleep disturbance, cognitive impairment, anxiety, and depression (8, 12–14), adversely affecting the quality of life (QOL) of patients with MS and burdening their families and society.

Physiotherapy rehabilitation (e.g., exercise therapy, multidisciplinary rehabilitation, and respiratory muscle training (RMT)) has beneficial effects on patients with MS (15–17). Since RMT can improve respiratory muscle function (18, 19), including it in rehabilitation programs for patients with MS is beneficial. RMT is defined as any intervention that improves the strength or endurance of inspiratory and/or expiratory muscles to enhance respiratory function; the common type of RMT is strength training, which includes endurance training (20). Strength training enhances the number and volume of muscle fibers, while endurance training increases the number of oxidative fibers and capillary density (21). An increase in respiratory muscle strength and endurance following RMT can reverse or delay the deterioration of respiratory muscle weakness, improve coughing ability, efficiently clear respiratory secretions, and reduce the sensation of dyspnea and fatigue (17). Therefore, RMT has been incorporated in the therapeutic strategies against neuromuscular disorders, such as Parkinson’s disease (22), spinal cord injury (23), stroke (24) and MS (25). Although the evidence is limited, RMT has been shown to improve lung volume and respiratory muscle strength in neuromuscular diseases (26, 27).

Despite the theoretical plausibility of RMT for patients with MS, there are limited data available to guide clinical practice because most trials lack control groups, and controlled studies involve small sample sizes, thereby limiting the level of evidence (16, 25, 28–30). In addition, evidence on the sustained effects of RMT remain limited, specifically regarding its long-term impacts on physical performance, fatigue, and QOL. Results from newly conducted clinical trials have recently been published (17, 31–35). However, the results of these studies are inconsistent. A recent systematic review and meta-analysis by Ferreira et al. (29) showed that RMT can improve ventilatory function and respiratory muscle strength in patients with neurodegenerative diseases, including MS and amyotrophic lateral sclerosis (ALS). However, MS and ALS differ significantly in pathogenesis, pathology, symptoms, disease course, prognosis, and patient needs. Forcing the combination of these two diseases in the analysis leads to ambiguous evidence, which may not only cause clinicians to misjudge the value of RMT for patients with MS but also fail to guide the development of specific training protocols targeted at MS. Herein, this systematic review and meta-analysis aimed to update the literature by incorporating the latest evidence and quantitative and qualitative analyses of the effectiveness of RMT interventions to determine their pooled effects on respiratory muscle strength, lung function, fatigue, exercise capacity, and QOL in patients with MS.

## 2 Methods

### 2.1 Protocol and registration

This review was registered in the International Prospective Register of Systematic Reviews (PROSPERO: CRD42023457664). For the type of included studies, we revised the inclusion criteria in the protocol to include only RCTs; ultimately, the inclusion criteria were expanded further to include randomized controlled trials (RCTs) and quasi-experimental (QE) studies. The guidelines of the Cochrane Collaboration were adopted to conduct this systematic review and meta-analysis and report it in the literature in accordance with the Preferred Reporting Items for Systematic Reviews and Meta-analysis recommendations (36).

### 2.2 Systematic literature search

Four electronic bibliographic databases (PubMed, Web of Science, Embase, and Cochrane) were systematically searched from inception to September 20, 2025. The search used a combination of medical subject headings (MeSH) and text words, such as “multiple sclerosis,” “disseminated sclerosis,” “sclerosis, multiple,” and “breathing exercises,” “breathing exercise*,” “breathing train*,” “inspiratory muscle training*,” “inspiratory muscle train*,” “inspiratory muscle strength,” “threshold load,” “threshold device,” “expiratory muscle training,” “expiratory muscle train*,” and “respiratory train*.” The detailed search strategy is presented in Supplementary Material S1. Other retrieval methods included literature tracking, contacting authors of studies for further information if the study had reported incomplete data, and searching for clinical trial registry platforms.

### 2.3 Inclusion and exclusion criteria

Articles that met the PICOS criteria were included. (1) Participants: We included only studies involving adult patients clinically diagnosed with MS. (2) Interventions: The experimental interventions consisted of RMT using resistance or endurance training devices, including isolated inspiratory muscle training (IMT), expiratory muscle training (EMT), or a combination of both. (3) Comparison: control interventions included non-training, sham training, and breathing exercises without devices. (4) Outcomes: Primary outcomes was respiratory muscle strength, included maximum inspiratory pressure (MIP), predicted MIP, maximum expiratory pressure (MEP), and predicted MEP, whereas secondary outcomes included lung function, exercise capacity, fatigue, QOL, adherence to treatment, and adverse events. Lung function included forced vital capacity (FVC), forced expiratory volume in first second (FEV_1_), vital capacity (VC), maximal voluntary ventilation (MVV), forced expiratory flow 25–75% (FEF_25–75%_), and et al. Assessment of exercise ability tools including but not limited to 6-min walking test (6MWT). Tools for assessing fatigue and QOL are not limited. The study reporting any one of the outcomes mentioned above was to be included. (5) Studies: We included both RCTs and QE, and conducted a quantitative synthesis. Crossover RCTs were considered using data up to crossover, if available. The exclusion criteria were as follows: (1) mixed participants with various neuromuscular diseases (e.g., amyotrophic lateral sclerosis); (2) primary intervention was RMT combined with physical exercise or RMT without load equipment, such as abdominal breathing, diaphragmatic breathing, and lip contraction breathing; (3) study protocols, duplicate publications, reviews, systematic evaluations, meta-analyses, conference abstracts, gray literature, or editorials; (4) non-English language literature.

### 2.4 Study selection and data extraction

Two authors (XYP and TOF) independently screened the retrieved studies by reading the titles, abstracts, and full texts, and excluded irrelevant literature and recorded the reasons for doing so. Two authors (XYP and TOF) independently extracted data from eligible studies based on predetermined Excel tables, which included study information (first author, publication year, country, and study design), patient characteristics (sample, age, sex, duration of MS, and expanded disability status scale), a brief description of the experimental and control interventions (devices, location, initial load, basis and frequency for adjusting load, sessions, frequency, duration, and supervision), and outcomes. Finally, two authors (XYP and TOF) independently extracted the study outcome data for quantitative analysis. The results of independent screening and extraction were cross checked by two reviewers (XYP and TOF). Disagreements were resolved through discussion.

### 2.5 Quality and risk of bias assessment

Two authors (XYP and TOF) independently evaluated the risk of bias in all included studies. Disagreements were resolved through discussion. The Cochrane Risk of Bias (RoB 2) Assessment Tool was used to assess risk bias in the RCTs (37). We assessed the risk of bias across the following domains: randomization process, deviations from intended interventions, missing outcome data, measurement of the outcome, and selection of the reported result. We used the RoB 2 Excel tool to complete the risk of bias assessment and judged each study as being at low risk, some concerns, or high risk. The Journal of Biomedical Informatics (JBI) quality evaluation tool for quasi-experimental studies includes nine items (38), which evaluate the overall quality of experimental studies from the causality of study variables, baseline, control, measurement of outcome indicators, and each item is assigned a qualitative assessment of either “yes,” “no,” “unclear,” or “inapplicable.”

### 2.6 Synthesis and statistical analysis

Review Manager 5.4 meta-analyses were carried out when comparable and single-construct outcome measures were available from a minimum of two studies; otherwise, they were synthesized qualitatively. When the group mean differences (MD) were not directly provided, we converted the median (range) format to the mean (standard deviation) (39). For the before-after studies, the outcomes were measured pre- and post-exercise; therefore, the results of pre-exercise measurements were selected for the meta-analysis to ensure comparability with those of other studies. The MDs served as the effect size when the studies employed the same tool for outcome assessment. Alternatively, standard mean differences (SMD) was used as the effect size when different tools were employed (40). All effect sizes are reported with 95% confidence intervals (CI). Cochran’s *Q* and *I*^2^ values were used to evaluate homogeneity among the studies (41). To assess heterogeneity among studies, we first inspected the distribution of effect measure point estimates and the overlap of their confidence intervals visually in the forest plot. Additionally, we employed the *I^2^* statistic to evaluate statistical consistency, which quantifies the proportion of total variation across studies that is due to between-study heterogeneity. We considered substantial statistical heterogeneity to be present when exceeded 50%. We explored the sources of heterogeneity through sensitivity analysis and subgroup analysis. Subgroup analysis and investigation of heterogeneity were performed to determine the influence of participant characteristics (such as age and MS type) and intervention-related factors (including dose of therapy and type of intervention) on the overall effects. We utilized the GRADE system to evaluate the quality of evidence for the specific outcomes in our review and summarized the key findings for the primary outcomes: MIP, predicted MIP, MEP, and predicted MEP.

## 3 Results

### 3.1 Study selection

**Figure 1:**
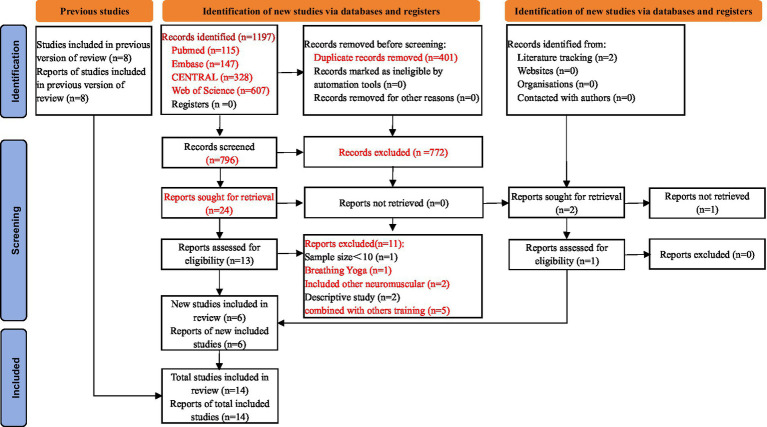
Flow diagram of the literature search.

A total of 1,197 articles were initially identified, and after removing duplicates, 796 relevant studies were selected. After excluding 772 patients during the initial screening by scanning titles and abstracts, we assessed the full text of 24 records for eligibility. Eleven records were excluded due to RMT combined with other training (42–46), MS combined with other neuromuscular disorders (47, 48), a breathing Yoga “Bhramary Pranayama” (49), a prospective descriptive study (50), a healthy control group (51) and a sample size below 10 (52). Literature tracking included two studies and one study that was not retrieved. Finally, 14 studies (17, 31–35, 53–60) met the inclusion criteria for our review, including eight RCTs (17, 34, 35, 55–58, 60) and six QE studies (31–33, 53, 54, 59) (two non-RCTs and four before-after trials). The selection procedure is illustrated in Figure 1.

### 3.2 Study characteristics

The details of the included studies are presented in Table 1. Fourteen studies were published between 1996 and 2022, with the number of study participants ranging from 15 to 77. Notably, 70% of the MS patients with MS were women. The duration of MS diagnosis varied greatly, with a mean age range of 8.43–27.6 years. Eight studies reported the type of MS in the participants; the degree of disability due to MS was mild to moderate (*n* = 8), severe (*n* = 5), and mixed (*n* = 1). Detailed outcomes of the included studies are presented in Supplementary Material S2.

**Table 1:** Characteristics of included studies.

Author (y)	Country	Study design	Sample (IG/CG)	Age (year)	Female (IG/CG)	Duration of MS (year)	Inclusion criteria for participant		EDSS	IG	CG
							Type of MS	Disability			
Smeltzer et al. (1996) (60)	USA	RCT	15 10/5	NR	8	14.1(6.6)	NR	Severe	Kurtzke disability scores 6.5–9.5	EMT	Sham training
Gosselink et al. (2000) (56)	Belgium	RCT	18 9/9	IG: 54 (13) CG:59(14)	9 3/6	IG: 24 (15) CG:31 (13)	NR	Severe	IG:8 (7–9) CG:8.5 (8–9.5)	EMT	Breathing exercise
Klefbeck and Hamrah (2003) (57)	Sweden	RCT	15 7/8	IG: 46 (37–49) CG:52.5 (38–61)	9 6/3	IG: 12 (3–19) CG: 20 (12–35)	Progressive MS	Severe	IG: 7.5 (6.5–8.0) CG: 8.0 (6.5–9.0)	IMT	Deep breathing
Chiara et al. (2006) (53)	USA	Before after trial	17	48.9 (7.61)	14	8.43 (6.17)	NR	Mild–moderate	3.62 (1.31)	EMT	Baseline
Chiara et al. (2007) (54)	USA	Before after trial	17	48.9 (7.61)	14	8.43 (6.17)	NR	Mild–moderate	3.62 (1.31)	EMT	baseline
Fry et al. (2007) (55)	USA	RCT	41 20/21	IG:50.0 (9.1) CG:46.2 (9.4)	31 18/13	NR	RR (*n* = 26), SP (*n* = 7), PP (*n* = 5), PR (*n* = 3)	Mild–moderate	IG: 3.96 (1.80) CG: 3.36 (1.47)	IMT	Not-training
Pfalzer and Fry (2011) (58)	USA	RCT	39 20/19	IG:49.6 (9.5) CG:46.0 (9.8)	31 18/13	NR	RR (*n* = 22), SP (*n* = 5), PP (*n* = 5), PR (*n* = 3), unknown (*n* = 4)	Mild–moderate	IG: 4.1 (1.9) CG: 3.2 (1.2)	IMT	Not-training
Ray et al. (2013) (59)	USA	Non-RCT	21 11/10	IG: 50.9 (5.7) CG: 56.2 (8.8)	16 9/7	IG:11.4 (8.3) CG:14.8 (8.3)	NR	Mild–moderate	IG: 3.2 (1.9) CG: 4.4 (2.1)	RMT	Not-training
Westerdahl et al. (2016) (35)	Sweden	RCT	48 23/25	IG:55 (12) CG:56 (9)	35 17/18	IG:24 (11) CG:23 (11)	RR (*n* = 20), SP (*n* = 26), PP (*n* = 2)	Mild–moderate	IG: 5.0 (3–7) CG: 4.5 (1.5–8)	EMT	Not-training
Silverman et al. (2017) (34)	USA	RCT	36 20/16	NR	31	NR	NR	Moderate	IG: 5.5 (1.5) CG: 5.48 (1.7)	EMT	Sham-training
Huang et al. (2020) (33)	Boston	Before after trial	36	60.5 (8.6)	27	27.6 (10.4)	Advanced MS	Severe	8.5 (0.4)	IMT	Baseline
Martin-Sanchez et al. (2020) (32)	Spain	Non-RCT	67 36/31	IG:50.03 (10.99) CG:53.06 (12.29)	41 22/19	IG: 16.50 (6.87) CG: 18.35 (7.85)	RR (*n* = 49), SP (*n* = 16), PP (*n* = 2)	Mixed	IG: 5.51 (2.31) CG: 5.21 (2.36)	IMT	Breathing exercise
Srp et al. (2021) (31)	Czech	Before after trial	26	52.7 (10.2)	17	23.3 (9.2)	RR (*n* = 11), PP (*n* = 4), SP (*n* = 11)	Severe	5.9 (0.6)	EMT	Baseline
Ghannadi et al. (2022) (17)	Iran	RCT	36 17/19	IG:36.47 (7.62) CG:39.36 (9.83)	27 13/14	NR	RR	Mild–moderate	IG: 3.52 (0.94) CG:3.07 (0.59)	IMT	Not-training

IG, intervention group; CG, control group; NR, not reported. RCT, randomized controlled trial; QE, quasi-experimental; EDSS, Expanded Disability Status Scale; Values expressed as Mean (standard deviation) or median (range). RR, relapsing remitting; SP, secondary progressive; PP, primary progressive; PR, progressive relapsing; IMT, inspiratory muscle training; EMT, expiratory muscle training.

### 3.3 Interventions

The details of the RMT protocols are shown in Table 2. Six studies (17, 32, 33, 55, 57) (four RCT_S_, two QE studies) performed isolated IMT with an initial threshold of 20–60%, adjusted weekly or biweekly based on the patient’s MIP, fatigue symptoms, or completion difficulty. The training duration ranged from 8 to 12 weeks, with sessions at least twice every other day and up to twice a day. Each session included three sets of 10–15 repetitions, except for Martin-Sanchez et al. (32), who performed 15 sets of 1 min each. Seven studies (31, 34, 35, 53, 56, 60) (four RCT_S_ and three QE studies) adopted a isolated EMT. Four studies (31, 34, 53, 56) used training loads greater than 60% of MEP. The training duration ranged from 5 to 12 weeks, with at least 5 days of training per week, and sometimes up to twice a day. Ray et al. (59) performed RMT using a T-shaped mouthpiece trainer with spring-loaded inlet and outlet valves for 30 min, 3 days per week, for 5 weeks.

**Table 2:** The detail of respiratory muscle training protocols.

Author (y)	Equipment	Location	Initial Load	Adjusted Load		Sessions	Frequency	Duration	Supervised
				Basis	Frequency				
Smeltzer et al. (1996) (60)	Threshold RMT devices	Home	MEP	Subject’s ability and difficulty	Fail completed, reduce 10% MEP	3 sets of 15 repetitions	2 session per day, daily	12 week	Patient daily record, home visits per week
Gosselink et al. (2000) (56)	Threshold RMT devices	NR	60% MEP	NR	NR	3 sets of 15 repetitions	2 session per day, daily	12 week	No reported
Klefbeck and Hamrah (2003) (57)	Threshold IMT	Home	40–60% MIP	MIP and RPE	Weekly	3 sets of 10 repetitions	twice every other day	10 week	Logbook, home visit
Chiara et al. (2006) (53)	Threshold trainer (16–160 cmH_2_O)	4 times at home	40% MEP the first week, 60% MEP the second week, and 80% MEP the 3–8 week.	MEP	Weekly	4 sets of 6 repetitions	5 d/wk	8 week	One supervised by investigator and 4 times with no supervise
Chiara et al. (2007) (54)	Threshold^®^PEP (16–160 cmH_2_O)	4 times at home	40% MEP the first week, 60% MEP the second week, and 80% MEP the 3–8 week.	MEP	Weekly	4 sets of 6 repetitions	5d/wk	8 week	One supervised by investigator and 4 times with no supervise
Fry et al. (2007) (55)	Threshold IMT	Home	30% MIP	Baseline MIP, RPE, symptoms	Weekly	3 sets of 15 repetitions	7 d/wk	10 week	Logbook and telephone
Pfalzer and Fry (2011) (58)	Threshold IMT	Home	30% MIP	Baseline MIP, RPE, symptoms	Weekly	3 sets of 15 repetitions	7 d/wk	10 week	Logbook and telephone
Ray et al. (2013) (59)	Resistive RMT of the inspiratory and expiratory muscles	One at laboratory, 2 times at home	Equal 25, 35, 40, 45, and 50% of MIP and MEP	MIP and MEP	Weekly	30 min/session	3d/wk	5 week	NR
Silverman et al. (2017) (34)	EMT 150	home	75% MEP	NR	weekly	5 sets of 5 repetitions	5 days/week	5-week	No reported
Westerdahl et al. (2016) (35)	A positive expiratory pressure device	Home	10–15 cmH_2_O	NR	NR	30 slow deep breaths	twice a day	8 week	Exercise diary, telephoned and letter
Huang et al. (2020) (33)	Threshold IMT	Home	30% MIP	Baseline MIP, RPE, symptoms	Weekly	3 sets of 15 repetitions	7 d/wk	10 week	Exercise log and rehabilitation aides
Martin-Sanchez et al. (2020) (32)	Threshold IMT	Home	First two-week 20% MIP, then 30% MIP	MIP	Biweekly	15 sets of one-minute	5 d/wk	12 week	Personal and telephone contact
Srp et al. (2021) (31)	Expiratory Muscle Trainer, EMT150 (30–150 cmH_2_O)	Home	60, 70, and 80% MEP for first, second and third month.	MEP	Monthly	5 sets of 5 forceful expirations	5 d/wk	12 week	Home therapy diary
Ghannadi et al. (2022) (17)	IMT device (POWER^®^ Breathe Classic)	Home	30% MIP	Breathing difficulty and symptoms	Weekly	3 sets of 15 repetitions	twice a day	8 week	Telephone, Logbook

RMT, respiratory muscle training; IMT, inspiratory muscle training; EMT, expiratory muscle training; MIP, maximum inspiratory pressure; MEP, maximum expiratory pressure; NR, not reported.

### 3.4 Risk of bias in included studies

**Figure 2:**
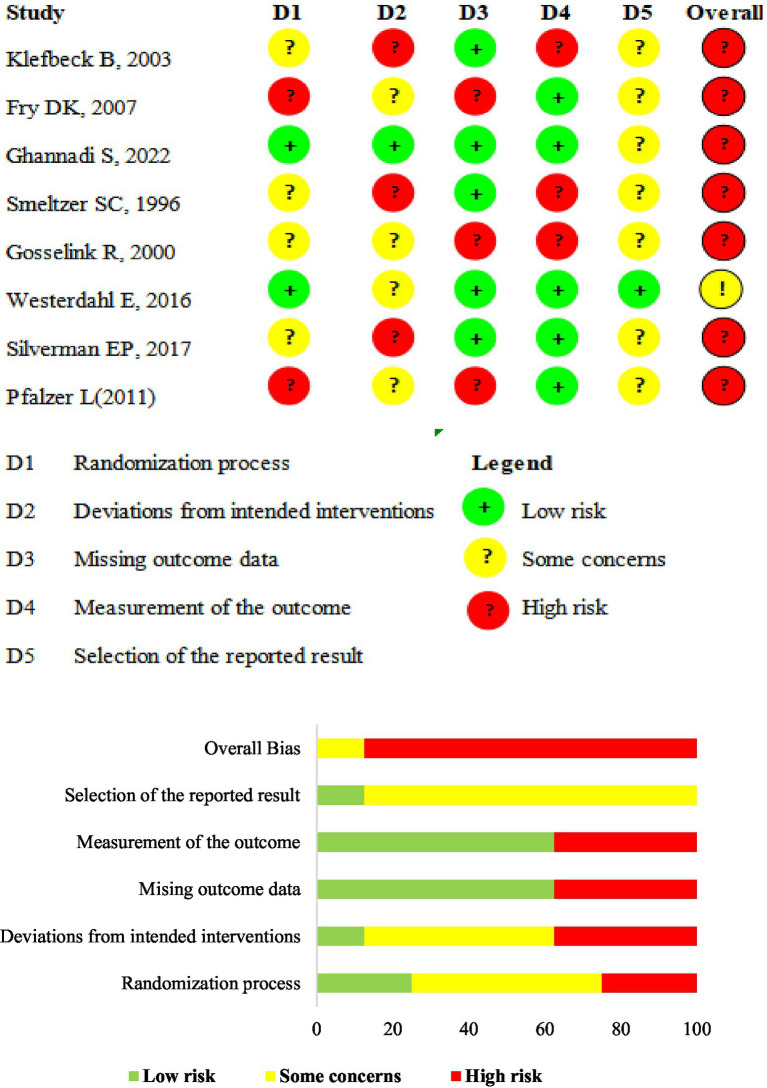
ROB 2.0 judgments according to domain and overall risk of bias for each study. Risk of bias summary. ROB 2.0 judgments according to the domain and overall risk of bias for each study.

In general, we assessed most of the studies (seven studies, 85.7%) as having a high risk of overall bias, while only one as having a concern for overall bias (35). We assessed the bias in the six included QE studies using the JBI Critical Appraisal Checklist for QE studies. We assessed most of the items as “Yes,” while 3 before-after trials were judged as “Not applicable” for item 2. For Item 6, Chiara et al. (53) and Chiara et al. (54) reported that several participants dropped out during the study, and these participants were not included in the final analysis. we registered a “No” response for such data points. Ray et al. (59) failed to report that the loss to follow-up was deemed ‘unclear.’ Table 3 presents details of the risk-of-bias assessment results. Figure 2 shows the results of the risk of bias assessment.

**Table 3:** JBI critical appraisal checklist for quasi-experimental studies.

Study ID	①	②	③	④	⑤	⑥	⑦	⑧	⑨
Martin-Sanchez et al. (2020) (32)	Y	Y	Y	Y	Y	Y	Y	Y	Y
Chiara et al. (2006) (53) Chiara et al. (2007) (54)	Y	NA	Y	Y	Y	N	Y	Y	Y
Ray et al. (2013) (59)	Y	Y	Y	Y	Y	U	Y	Y	Y
Srp et al. (2021) (31)	Y	NA	Y	Y	Y	Y	Y	Y	Y
Huang et al. (2020) (33)	Y	NA	Y	Y	Y	Y	Y	Y	Y

① Is it clear in the study what is the ‘cause’ and what is the ‘effect’?

② Were the participants included in any comparisons similar?

③ Were the participants included in any comparisons receiving similar treatment/care, other than the exposure or intervention of interest?

④ Was there a control group?

⑤ Were there multiple measurements of the outcome both pre and post the intervention/exposure?

⑥ Was follow up complete and if not, were differences between groups in terms of their follow up adequately described and analyzed?

⑦ Were the outcomes of participants included in any comparisons measured in the same way?

⑧ Were outcomes measured in a reliable way?

⑨ Was appropriate statistical analysis used? Y: Yes; N: No; U: Unclear; NA: Not applicable.

### 3.5 Effects of interventions

#### 3.5.1 MIP

**Figure 3:**
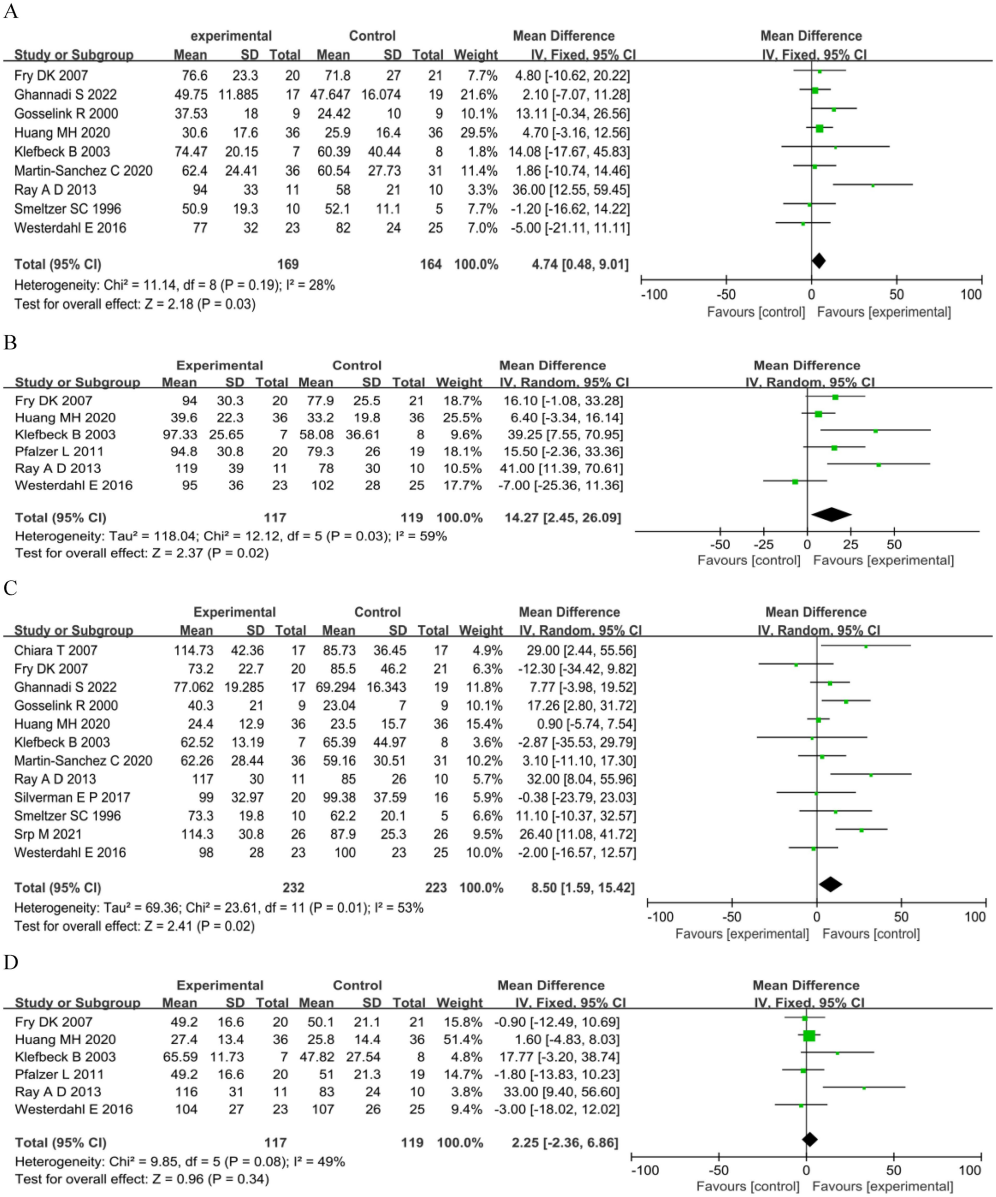
Respiratory muscle strength. **(A)** Maximum inspiratory pressure (cmH2O). **(B)** Predicted maximum inspiratory pressure MIP (%). **(C)** Maximum expiratory pressure (cmH2O). **(D)** Predicted maximum expiratory pressure (%).

The MIP was reported in nine studies: six RCTs (17, 35, 55–57, 60) and three QE studies (32, 33, 59). The training led to a significant increase in MIP compared to control interventions (MD 4.74 cmH_2_O, 95%CI 0.48–9.01, *p* = 0.03, *I*^2^ = 28%; Figure 3A). Although we noted moderate heterogeneity in the included studies, small-study effects may be present; we downgraded the outcome for risk of bias and publication bias. We rated the quality of the evidence as low. Subgroup analysis was performed according to the study type, intervention type, degree of disability, and intervention period. The results showed no significant differences between the groups (Table 4 and Supplementary Material S3).

**Table 4:** Subgroup analyses of MIP and MEP.

Outcomes	Item	No. of studies	Sample	Heterogeneity		meta-analysis	*p*-value
				*I* ^2^	*P*-value		
MIP (cmH_2_O)							
Type of studies							
RCT		6 (17, 35, 55–57, 60)	173	0%	0.56	3.50 (−2.21, 9.21)	0.23
QE		3 (31, 32, 59)	160	71%	0.03	10.31 (−3.93, 24.54)	0.16
Type of interventions							
IMT		5 (17, 32, 33, 55, 57)	231	0%	0.95	3.71 (−1.31, 8.74)	0.15
EMT		3 (35, 56, 60)	81	41%	0.18	3.54 (−5.04, 12.12)	0.42
RMT		1 (59)	21	–	–		
Disability level							
Mild–moderate		4 (17, 35, 55, 59)	146	65%	0.04	6.82 (−5.93, 19.57)	0.29
Severe		4 (33, 56, 57, 60)	120	0%	0.52	5.85 (−0.25, 11.95)	0.06
Mixed		1 (32)	67	–	–		
Intervention duration							
>8 week		6 (32, 33, 55–57, 60)	228	0%	0.77	5.06 (−0.11, 10.23)	0.06
≤8 week		3 (17, 35, 59)	105	77%	0.01	8.58 (−9.98, 27.14)	0.36
Predicted MIP (%)							
Type of studies							
RCT		4 (35, 55, 57, 58)	143	59%	0.06	13.26 (−2.55, 29.07)	0.10
QE		2 (33, 59)	93	79%	0.03	20.76 (−12.66, 54.17)	0.22
Type of interventions							
IMT		4 (55, 57, 58)	167	33%	0.22	11.61 (4.17, 19.05)	0.002
EMT		1 (35)	48	–	–		
RMT		1 (59)	21	–	–		
Disability level							
Mild–moderate		4 (35, 55, 58, 59)	149	63%	0.04	14.08 (−2.41, 30.58)	0.09
Severe		2 (33, 57)	87	73%	0.05	19.22 (−12.19, 50.63)	0.23
Intervention duration							
>8 week		4 (33, 55, 57, 58)	167	33%	0.22	13.74 (3.58, 23.90)	0.008
≤8 week		2 (35, 59)	93	79%	0.03	20.76 (−12.66, 54.17)	0.22
MEP (cmH_2_O)							
Type of studies							
RCT		7 (17, 34, 35, 55–57, 60)	209	14%	0.33	4.96 (−2.22, 12.14)	0.18
QE		5 (31–33, 54, 59)	246	76%	0.002	15.55 (1.76, 29.35)	0.03
Type of interventions							
IMT		5 (17, 32, 33, 55, 57)	231	0%	0.60	1.70 (−3.44, 6.83)	0.52
EMT		6 (31, 34, 35, 54, 56, 60)	203	50%	0.07	13.05 (5.82,20.27)	0.0004
RMT		1 (59)	21	–	–		
Disability level							
Mild–moderate		6 (17, 34, 35, 54, 55, 59)	216	57%	0.04	7.46 (−4.43, 19.36)	0.22
Severe		5 (31, 33, 56, 57, 60)	172	66%	0.02	11.35 (−0.51, 23.21)	0.06
Mixed		1 (32)	67	–	–		
Intervention Duration							
>8 week		7 (31–33, 55–57, 60)	280	59%	0.02	7.38 (−1.83, 16.60)	0.12
≤8 week		5 (17, 34, 35, 54, 59)	175	53%	0.07	10.71 (−1.51, 22.94)	0.09
Predicted MEP (%)							
Type of studies							
RCT		4 (35, 55, 57, 58)	143	0%	0.39	0.38 (−6.52, 7.29)	0.91
QE		2 (33, 59)	93	84%	0.01	15.16 (−15.32, 45.65)	0.33
Type of Interventions							
IMT		4 (33, 55, 57, 58)	167	0%	0.43	1.47 (−3.48, 6.42)	0.56
EMT		1 (35)	48	–	–		
RMT		1 (59)	21	–	–		
Disability level							
Mild–moderate		4 (35, 55, 58, 59)	149	61%	0.05	3.59 (−8.10, 15.28)	0.55
Severe		2 (33, 57)	87	52%	0.15	6.48 (−8.07, 21.02)	0.38
Intervention duration							
>8 week		4 (33, 55, 57, 58)	167	0%	0.43	1.47 (−3.48, 6.42)	0.56
≤8 week		2 (35, 59)	69	84%	0.01	13.80 (−21.40, 49.00)	0.44

MIP, maximum inspiratory pressure; MEP, maximum expiratory pressure; RCT, randomized controlled trial; QE, quasi-experimental; RMT, respiratory muscle training; IMT, inspiratory muscle training; EMT, expiratory muscle training.

For predicted MIP (33, 35, 54, 57–59), a significant benefit in the experimental arm (MD 14.27, 95%CI 2.45–26.09, *p* = 0.02; Figure 3B) was observed. We noted high heterogeneity in the included studies; we downgraded the outcome for risk of bias, imprecision, and publication bias, and rated the quality of evidence as very low. Subgroup analysis showed that IMT and intervention for > 8 weeks were significant (MD 11.61, 95%CI 4.17–19.05, *p* = 0.002), (MD 13.74, 95%CI 3.58–23.90, *p* = 0.008) (Table 4 and Supplementary Material S3).

#### 3.5.2 MEP

Twelve studies assessed PEmax: seven RCTs (17, 34, 35, 55–57, 60) and five QE studies (31–33, 54, 59). Meta analysis showed a significant summary effect size on MEP (MD 8.50 cmH_2_O, 95%CI 1.59–15.42, *p* = 0.02; Figure 3C). We noted high heterogeneity in the included studies, with potential small-study effects; therefore, we downgraded the outcome for risk of bias and publication bias and rated the quality of evidence as low. Subgroup analysis showed that only QE study and EMT were significant (MD 15.55 cmH_2_O, 95%CI 1.76–29.35, *p* = 0.03; MD 13.05 cmH_2_O, 95%CI 5.82–20.27, *p* = 0.0004; Table 4 and Supplementary Material S3).

For predicted MEP (33, 35, 54, 57–59), no significant benefit in the experimental arm (MD 2.25, 95%CI −2.36–6.86, *p =* 0.34; Figure 3D) was observed. We noted a moderate heterogeneity between the trials; small-study effects may be present. Therefore, we downgraded the outcome for risk of bias and publication bias and rated the quality of evidence as low. The subgroup analysis showed no significant differences (Table 4 and Supplementary Material S3).

#### 3.5.3 Lung function

**Figure 4:**
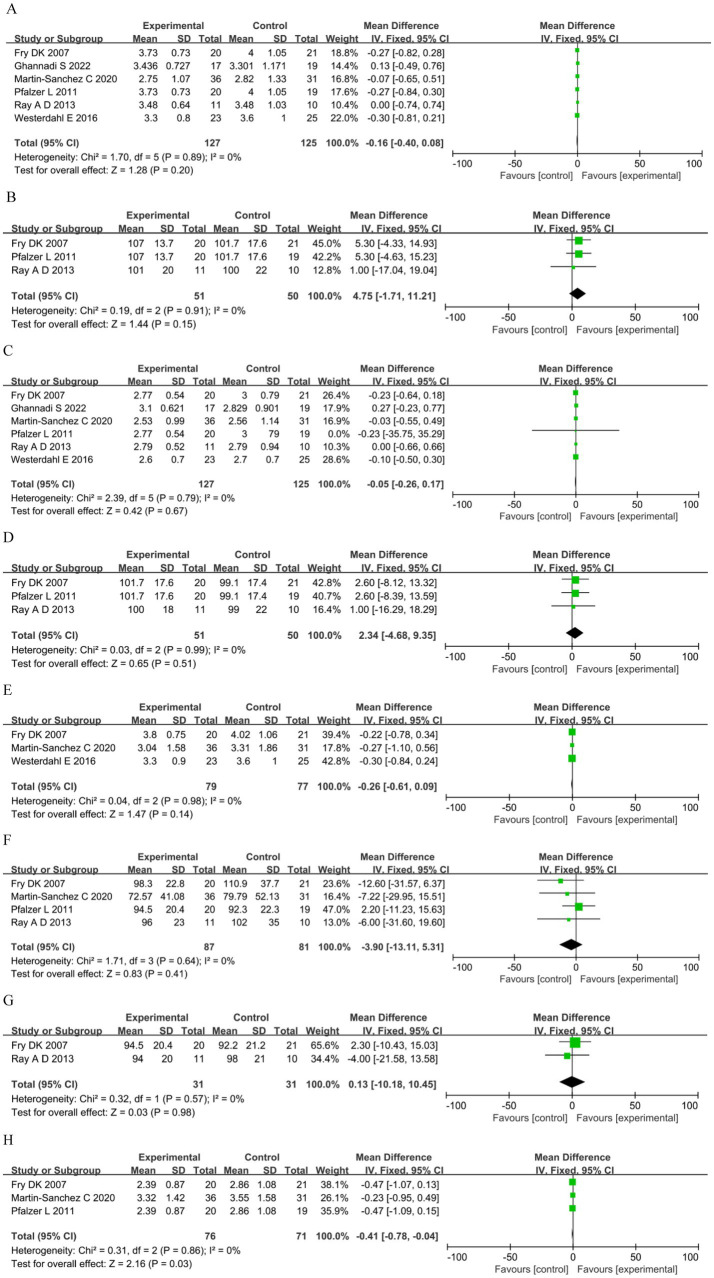
Lung function. **(A)** Forced vital capacity (L). **(B)** Predicted forced vital capacity (%). **(C)** Forced expiratory volume in the first second (L). **(D)** Predicted forced expiratory volume in the first second (%). **(E)** Vital capacity (L). **(F)** Maximal voluntary ventilation (L). **(G)**: Predicted maximal voluntary ventilation (%). **(H)** Forced expiratory flow 25–75% (FEF25-75%).

Meta-analysis comparing RMT versus control under a fixed-effect model showed no significant summary effect size on FVC (MD −0.16 L, 95%CI 0.40–0.08, *p =* 0.20), predicted FVC (MD 4.75, 95%CI −1.71–11.21, *p =* 0.15), FEV_1_ (MD −0.05 L, 95%CI −0.26–0.17, *p =* 0.67), predicted FEV_1_ (MD 2.34, 95%CI −4.68–9.35, *p =* 0.51), VC (MD −0.26 L, 95%CI −0.61–0.09, *p =* 0.14), MVV (MD −3.90, 95%CI −13.11–5.31, *p =* 0.41), or predicted MVV (MD 0.13, 95%CI −10.18–10.45, *p =* 0.98); only FEF_25–75%_ was significant (MD −0.41, 95%CI −0.78–−0.04, *p =* 0.03) (Figure 4).

#### 3.5.4 Fatigue and exercise capacity

**Figure 5:**
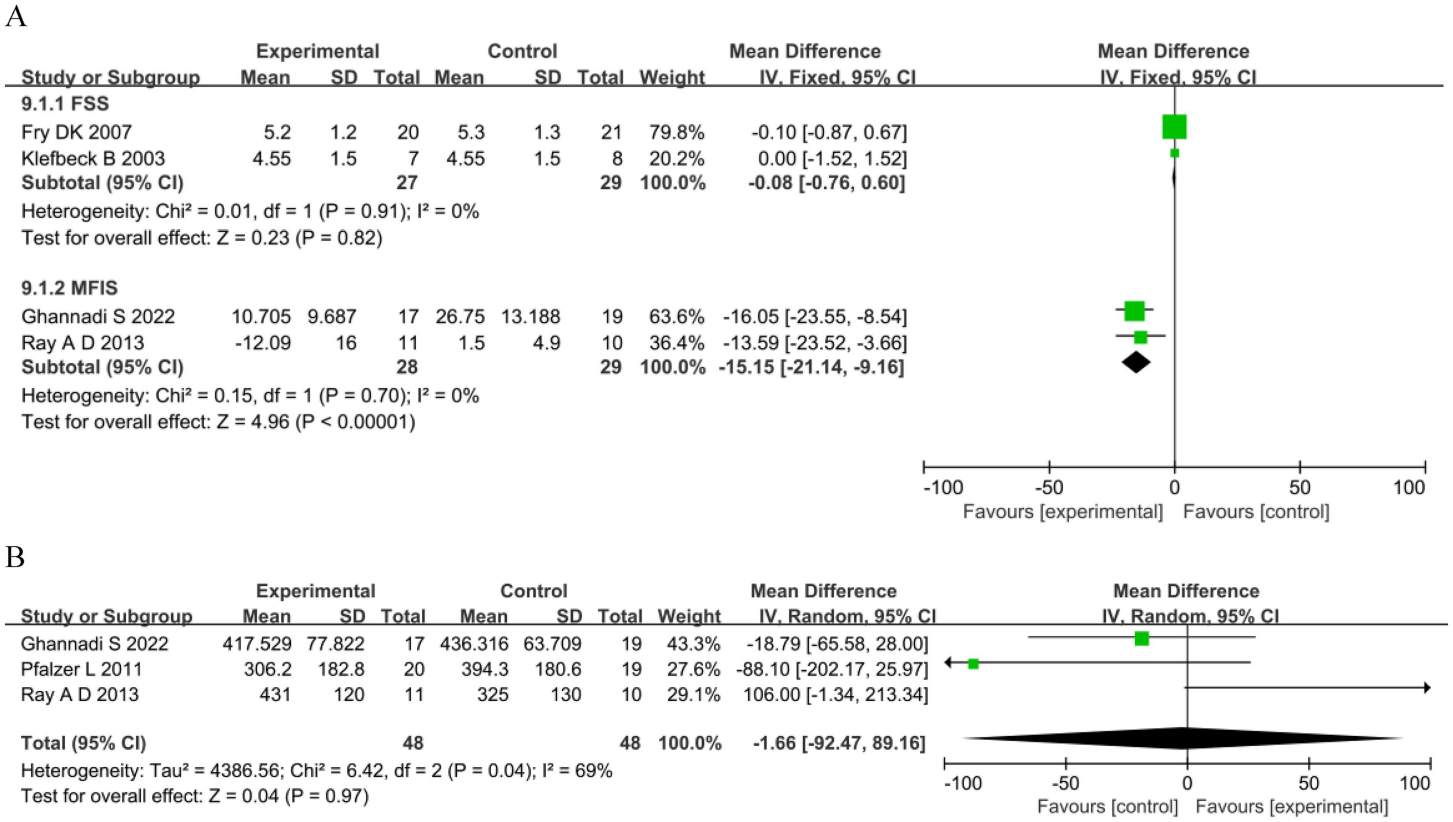
Other outcomes. **(A)** Fatigue. **(B)** 6-minute walking test (m).

Four studies assessed fatigue: two (49, 51) used the Fatigue Severity Scale (FSS) and two (17, 53) used the Modified Fatigue Impact Scale (MFIS). The analysis showed no significant differences in the FSS between the groups. However, for MFIS in the RMT group was significantly reduced compared with the control group (MD −15.15, 95%CI −21.14–−9.16, *p* < 0.00001) (Figure 5A). Exercise capacity was reported in three studies (17, 58, 59) as meters walked during the 6MWT. The analysis showed no significant differences between groups (MD −1.66, 95%CI −92.47–89.16, *p =* 0.97; Figure 5B).

#### 3.5.5 QOL

Four trials reported QOL results. Two trials reported the 36-item short-form (SF) (17, 59), while one (59) reported that the control group showed decreased emotional well-being and general health. Ghannadi et al. (17) found that the 36-item SF score significantly improved in the intervention group (*p* < 0.005). One trial reported that the 12-item SF (32) did not significantly change between the two groups following intervention. One trial reported the Euro QoL EQ-5D visual analog scale (35), which was not significantly different (*p* < 0.136). Due to the small number of studies, a meta-analysis of the QOL measures was not possible.

#### 3.5.6 Adherence to treatment

Treatment adherence was not objectively measured in any of the studies. However, four studies (33, 54, 55, 58) reported compliance with IMT as an indirect indicator of adherence. Pfalzer et al. (58) reported an adherence to the IMT training protocol ranging from 76.25 to 83.50%. Fry et al. (55) reported an average adherence rate of 81% to an IMT training protocol. Chiara et al. (54) showed that training compliance, measured by participants’ logs, ranged from 90 to 100%. Huang et al. (33) reported that participants completed 47 ± 29% of the prescribed repetitions during a 10-week IMT training.

#### 3.5.7 Adverse events

Fry et al. (55) reported that all participants tolerated the exercise training program, except for one who complained of light-headedness during the initial training session. To resolve the light-headedness of the participant, the pressure resistance was reduced by 2 cm H_2_O. Huang et al. (33) reported that participants tolerated inspiratory exercises well without experiencing adverse events. Westerdahl et al. (35) reported that 17% of the patients experienced some discomfort.

## 4 Discussion

This systematic review and meta-analysis summarized evidence from 14 studies involving 373 participants to identify the effects of respiratory muscle training interventions among patients with MS. The pooled results showed that the RMT program was an effective intervention to improve MIP, predicted MIP, MEP, and fatigue, but showed no significant effect on predicted MEP, lung function, exercise capacity, and QOL. This result is not only consistent with the pathophysiological characteristics of respiratory dysfunction in patients with MS but also provides reference suggestions for clinicians to develop rehabilitation programs.

Our study found that RMT can significantly improve respiratory muscle strength and alleviate fatigue in patients with MS. The pathological basis of MS is demyelinating lesions in the central nervous system, which can involve the motor nerves innervating the diaphragm and intercostal muscles, leading to a decrease in respiratory muscle strength and endurance (6). This is the primary cause of respiratory fatigue and weak cough in affected patients. Respiratory muscle training (such as threshold load training and resistance training) stimulates the adaptive hyperplasia of muscle fibers through load application and enhances the efficiency of neuromuscular recruitment, thereby improving the active contraction ability of respiratory muscles (25). Consequently, it can significantly MIP, which reflects the strength of inspiratory muscles (primarily the diaphragm) and MEP, which reflects the strength of expiratory muscles (mainly the internal intercostal muscles and abdominal muscles). This change is highly consistent with the root cause of respiratory dysfunction in patients with MS.

However, this study found that RMT did not significantly improve preMEP in MS patients. The predicted MEP is based on the standardized reference values of age, sex, and height. The actual value of MEP reflects the current expiratory muscle active strength, and the percentage of the actual value of MEP to the predicted value reflects the gap between the actual and potential functional capacity. A percentage below 80% indicates insufficient expiratory muscle strength. RMT improves the actual value of MEP in patients with MS, but does not significantly increase the percentage of the actual value of MEP to the predicted value. This suggests that RMT can improve the current respiratory muscle strength of MS patients, but cannot exceed the theoretical upper limit determined by individual physiological conditions (e.g., muscle fiber volume and nerve supply).

A total of four studies (17, 49, 51, 53) reported the impact of RMT on the level of fatigue in patients with MS. Sadr et al. (49) and Smeltzer and Lavietes et al. (51) reported that IMT did not alleviate fatigue when they used independent IMT formats. Chiara et al. (53) employed RMT, Ghannadi et al. (17) used independent IMT formats, both of which resulted in a marked improvement in the fatigue in MS patients. The underlying reason for these conflicting results may be the training method, study subjects, and fatigue assessment tools used in each study. The main reason is probably due to differences in training plans, study participants, and evaluation tools. First, the training protocol: Chiara et al. (53) used inspiratory and expiratory muscle training, which included the inspiratory muscles that are earliest affected in MS patients. Ghannadi et al. (17) used high-frequency, individually adjusted, independent IMT, while Sadr et al. (49) used single-IMT with no individualized intensity adjustment. Smeltzer and Lavietes (51) had low training frequency and low single-session training volume, which made it difficult to effectively stimulate the respiratory muscles. Secondly, differences in study subject characteristics: Chiara et al. (53) and Ghannadi et al. (17) enrolled patients with mild to moderate disability (Expanded Disability Status Scale [EDSS] ≤ 6.5), where fatigue primarily resulted from respiratory muscle weakness. Sadr et al. (49) enrolled patients with 73.9% experiencing brain dysfunction, Smeltzer and Lavietes (51) enrolled patients with EDSS ≥ 6.5, predominantly wheelchair-dependent or bedridden, fatigue triggers included not only respiratory muscle weakness but also a multifactorial overlap of central fatigue and psychological factors. Finally, fatigue assessment tools are different. Chiara et al. (53) and Ghannadi et al. (17) used the multidimensional MFIS scale, which can distinguish respiratory-related fatigue. Sadr et al. (49) and Smeltzer and Lavietes (51) used the unidimensional FSS scale, which cannot discriminate fatigue triggers and exhibits a ceiling effect at high baseline fatigue levels, leading to differing outcomes. It is recommended that clinicians develop personalized rehabilitation protocols based on the disability level and fatigue triggers of MS patients, rather than relying on a single training program.

This study found that RMT did not significantly improve lung function in MS patients. Lung function in MS patients is not solely dependent on respiratory muscle strength but is also related to lung tissue elasticity, airway patency, and thoracic compliance. The respiratory impairment in MS patients is primarily due to abnormal innervation of respiratory muscles, rather than lung tissue destruction (such as emphysema or pulmonary fibrosis) or airway obstruction (such as asthma or COPD). MS patients exhibit no significant structural damage to lung tissue, and thoracic mobility does not undergo fundamental changes due to training. Pre-improvement lung ventilation function shows no marked decline in MS patients. Consequently, post-training lung ventilation indicators reflecting lung volume and airway patency, such as FVC and FEV_1,_ did not significant increased. Notably, the study findings revealed a significant decrease in FEF25-75%, suggesting vigilance is warranted regarding training-related airway dynamics or measurement bias. FEF25-75% reflects expiratory flow in small and medium airways and serves as a sensitive indicator of their patency. A significant decline in this parameter necessitates careful analysis in the clinical context.

Several systematic reviews have assessed the effects of RMT interventions on patients with MS (16, 25, 28–30). The agreements and disagreements we observed when comparing these published reviews are shown in Supplementary Material S4. The current systematic review included six new studies (17, 31–34, 54) (two RCTs and four QEs). Martín-Valero et al. (30) included five RCTs pooled by meta-analysis, revealing that IMT and EMT were effective in improving MIP, MEP, FVC, pulmonary dysfunction index, and quality of life. Campbell et al. (16) reported that physiotherapy interventions, including one study on IMT with progressive MS, significantly improved MIP and MEP. Ferreira et al. (29) enrolled patients with MS and amyotrophic lateral sclerosis; six of them had MS. The authors showed that RMT improved MIP and MEP. Rietberg et al. (25) included six RCTs comprising 195 participants with MS, two with IMT, three with EMT, and one with regular breathing exercise. Pooled and analyzed data of five trials with 137 MS indicated that IMT was an effective post-intervention for improving predicted MIP, whereas EMT showed no significant effects. Due to the low number of studies included, subgroup analyses were not performed. The study by Mutluay et al. (42) was not included in our systematic review since the intervention was a breathing-enhanced upper extremity exercise. One recent review (28) reported that 11 respiratory rehabilitation interventions, including RMT and deep-breathing exercises, were retained for review; the authors showed that RMT could improve MIP in MS, and that lung volume recruitment could slow the decline in vital capacity. RCTs, non-RCTs, and observational studies were included in their review. Thus, existing reviews do not provide sufficient evidence regarding the effects of RMT interventions on maximal inspiratory pressure, lung function, fatigue, exercise capacity, QOL, or adverse events. Additionally, we performed subgroup analyses according to study type, intervention type, disability degree, and intervention period, which were not included in the previous systematic review due to the limited number of trials. Our study also included more outcomes, such as exercise capacity and QOL.

The RMT interventions improved MIP, predicted MIP, and MEP; these findings were inconsistent with those of previous reviews. Although a Meta-analysis counted IMT and EMT separately, no definitive evidence was found due to the limited sample size (25). Our study attempted to combine IMT with EMT; subgroup analyses were performed according to the types of intervention. Notably, there were fewer significant outcomes in the subgroup analyses. IMT improved predicted MIP, and EMT improved MEP. Martin-Sanchez et al. (32) reported that an IMT with low resistance improved MIP and MEP by 51 and 36%, respectively. One possible reason could be that patients with MS have lower MIP and MEP values; the muscles primarily affected are the expiratory muscles, especially for severely impaired patients, and finally, the inspiratory muscles (5, 59, 61). Additionally, our review included different types and intensities of RMT and was unable to determine the optimal training prescriptions for frequency, intensity, or duration.

In summary, when developing rehabilitation protocols for MS patients, clinicians should fully consider the specificity of respiratory muscle training. For MS patients with impaired respiratory muscle function, targeted respiratory muscle training should be intensified. For MS patients with concomitant pulmonary ventilation dysfunction and/or decreased limb mobility, comprehensive rehabilitation training protocols should be established. Concurrently, clinicians should regularly monitor changes in MIP and MEP in MS patients to detect early declines in respiratory muscle function. Timely respiratory muscle training interventions are crucial to prevent serious complications such as subsequent pulmonary infections and respiratory failure.

## 5 Limitations

This study had some limitations. First, our study both included RCTs and QE studies. Since MS is not a common neurological disease (with a global prevalence of 35.9 per 100,000 population in 2020) and is associated with varying degrees of physical disability, it has a long disease course and the condition may change at any time (1). Additionally, RMT requires MS patients to modify their existing lifestyles, which results in difficulties in recruiting participants for clinical trials. Consequently, the number of RCT is limited, especially for MS patients with severe disability. In this study, over 70% of the included MS patients had mild to moderate disability. For MS patients with severe disability, most studies adopted before after trial. A total of 4 before-after trials (31, 33, 53, 54) were included in this study, among which 64.6% of the subjects were MS patients with severe disability, accounting for 56.36% of all MS patients with severe disability included in the entire study. Although before-after trials do not have an independent parallel control group, the self-comparison between “pre-intervention baseline and post-intervention” can effectively reflect the effect of respiratory muscle training on MS patients. If these studies were excluded, it would lead to a lack of data on MS patients with severe disability and significantly reduce the representativeness of the meta-analysis results for MS patients throughout the entire disease course. However, before-after trials cannot rule out the impact of confounding factors such as natural disease course, repeated measurement effects, and placebo. It is recommended that more RCTs targeting MS patients with severe disability be conducted in the future to provide evidence of a higher level. Although subgroup analyses of the primary outcome were performed, the quality of evidence remained limited. Second, the quality of the included studies, several of which were high-risk studies, compromised the overall quality of the evidence. The included patients with MS had a wide age range and different degrees of disability, which might have affected the results. Moreover, although our study did not calculate effect sizes separately for IMT, EMT, and RMT, we performed subgroup analyses based on different interventions. We found high variability in the protocols used for the RMT programs, especially regarding external load, which ranged from 20 to 75% PImax/PEmax, and intervention durations ranged from 5 to 12 weeks, variable protocols weakens the certainty of conclusions. Finally, despite conducting a thorough search and including additional research, such as the six new studies, the RMT evidence presented in this study remains insufficient for patients with MS. In addition, the GRADE ratings for the primary outcome measures in this study were all low or very low, we included only English-language literature and excluded gray literature, which may introduce bias.

## 6 Conclusion

Via a comprehensive search and integration of evidence, this systematic review and meta-analysis revealed that RMT interventions are effective in improving MIP, predicted MIP, MEP, and fatigue; however, these interventions had no significant effect on predicted MEP, lung function, exercise capacity, and QOL. Therefore, available evidence remains insufficient to support the effects of RMT. This necessitates well-designed randomized controlled trials to explore the effects of different intervention types, MS types, and interventions.

## Data Availability

The raw data supporting the conclusions of this article will be made available by the authors, without undue reservation.
