# Supplementary material for: Effectiveness of respiratory muscle training in adults with multiple sclerosis: a systematic review and meta-analysis
Source: Front Neurol. 2025 Oct 23;16:1665651. doi: 10.3389/fneur.2025.1665651 (PMC12590560; doi:10.3389/fneur.2025.1665651)
Supplement: Supplementary file 1 [file Table_1.docx]

**Effect of** **respiratory muscle training in adults with multiple sclerosis: A systematic review and meta-analysis**

**Supplement table 1** : **Search strategy**

| **1.Pubmed（115）** | | |
| --- | --- | --- |
| #1 | "Breathing Exercises"[Mesh] | 4,748 |
| #2 | ((((((((((((Breathing Exercise*[Title/Abstract]) OR (Breathing Train*[Title/Abstract]))OR (Inspiratory Muscle Training[Title/Abstract])) OR (Inspiratory Muscle Train*[Title/Abstract])) OR (Inspiratory Muscle Strength[Title/Abstract])) OR (threshold load[Title/Abstract])) OR (threshold device[Title/Abstract])) OR (Expiratory muscle training[Title/Abstract])) OR (Expiratory muscle train*[Title/Abstract])) OR (respiratory train[Title/Abstract])) OR (IMT[Title/Abstract])) OR (RMT[Title/Abstract])) OR (EMT[Title/Abstract]) | 57,847 |
| #3 | #1 OR #2 | 60,881 |
| #4 | "Multiple Sclerosis"[Mesh] | 76,079 |
| #5 | ((Sclerosis,Multiple[Title/Abstract]) OR (Disseminated Sclerosis[Title/Abstract])) OR (MS[Title/Abstract]) | 518,166 |
| #6 | #4 OR #5 | 549,484 |
| #7 | (randomized controlled trial[Publication Type]) OR (controlled clinical trial[Publication Type]) | 738,676 |
| #8 | ((((randomized[Title/Abstract]) OR (placebo[Title/Abstract])) OR (clinical trials[Title/Abstract])) OR (randomly[Title/Abstract])) OR (trial[Title/Abstract]) | 1,962,829 |
| #9 | #7 OR #8 | 2,136,540 |
| #10 | #3 AND #7 AND #9 | 115 |
| **2.Embase（147）** | | |
| #1 | ("multiple sclerosis" or "disseminated sclerosis" or MS).af. | 1,007,875 |
| #2 | ('breathing exercise*' or 'breathing train*' or 'inspiratory muscle train*' or 'inspiratory muscle strength' or 'threshold load' or 'expiratory muscle train*' or 'respiratory train').af. | 17,409 |
| #3 | (randomized controlled trial or controlled clinical trial or random* or clinical trials).af. | 3,683,630 |
| #4 | #1 AND #2 AND #3 | 147 |
| **3.CENTRAL（328）** | | |
| #1 | MeSH descriptor: [Breathing Exercises] this term only | 1,476 |
| #2 | (Breathing Exercise*):ti,ab,kw OR (Breathing Train*):ti,ab,kw OR (Inspiratory Muscle Training):ti,ab,kw OR (Inspiratory Muscle Train*):ti,ab,kw OR (Inspiratory Muscle Strength):ti,ab,kw | 12,025 |
| #3 | (expiratory muscle training):ti,ab,kw OR (expiratory muscle train*):ti,ab,kw OR (respiratory train):ti,ab,kw | 9,060 |
| #4 | #1 OR #2 OR #3 | 17,728 |
| #5 | (multiple sclerosis):ti,ab,kw OR (disseminated sclerosis):ti,ab,kw OR (MS):ti,ab,kw | 31,834 |
| #6 | #4 AND #5 | 328 |
| **4.web of science（607）** | | |
| #1 | ((((((((((((TS=(Breathing Exercise*)) OR TS=(Breathing Exercise*)) OR TS=(Inspiratory Muscle Training)) OR TS=(Inspiratory Muscle Train*)) OR TS=(Inspiratory Muscle Strength)) OR TS=(threshold load)) OR TS=(threshold device)) OR TS=(Expiratory muscle training)) OR TS=(Expiratory muscle train*)) OR TS=(respiratory train)) OR TS=(IMT)) OR TS=(EMT)) OR TS=(RMT) | 813,252 |
| #2 | (((TS=(Multiple Sclerosis)) OR TS=(Sclerosis,Multiple)) OR TS=(Disseminated Sclerosis)) OR TS=(MS ) | 1,323,593 |
| #3 | ((((((TS=(randomized controlled trial)) OR TS=(controlled clinical trial)) OR TS=(randomized)) OR TS=(placebo)) OR TS=(clinical trials)) OR TS=(randomly)) OR TS=(trial) | 4,552,279 |
| #4 | #1 AND #2 AND #3 | 607 |
