# Supplementary material for: Effectiveness of respiratory muscle training in adults with multiple sclerosis: a systematic review and meta-analysis
Source: Front Neurol. 2025 Oct 23;16:1665651. doi: 10.3389/fneur.2025.1665651 (PMC12590560; doi:10.3389/fneur.2025.1665651)
Supplement: Supplementary file 2 [file Table_2.docx]

**Effect of** **respiratory muscle training in adults with multiple sclerosis: A systematic review and meta-analysis**

**Supplement table 2: detail outcomes of included studies**

| **Detail outcomes of included studies** | | | |
| --- | --- | --- | --- |
| **Author (y)** | **Outcomes measures** | **Findings** | |
|  |  | **IG** | **CG** |
| Smeltzer SC et al.,  1996 (1) | MIP  MEP | MIP (cmH_2_O): Pre 47.6(19.3); post no significant changes  MEP (cmH_2_O): Pre 53.6(14.9); post increased 19.4(9.9) cmH_2_0; *p* = 0.003 | No significant changes |
| Gosselink R et al.,  2000 (2) | MIP  MEP  FVC  PI | MIP (cmH_2_O): Pre 27(18); post 39(41); *p* < 0.05  MEP (cmH_2_O): Pre 31(21); post increased 8(14) cmH_2_0, *p* = 0.08  FVC (% predicted) :no significant changes  PI: improved significantly compared with baseline, *p* < 0.05 | No significant changes |
| Klefbeck B et al.,  2003 (3) | MIP  MEP  FSS  Borg RPE scale | MIP (cmH_2_O): Pre 42(28-74); post 67(55-110), *P* < 0.008  MIP(% predicted): Pre 58(38-93); post 92(67-137)  MEP (cmH_2_O): Pre 46(36-58); post 63(44-80), *P* < 0.02  MEP(% predicted): Pre 49(39-58); post 68(47-79)  FSS: Pre 5.3 (2.5-5.9); post 5.2 (1.8-5.9)  Borg RPE scale: Pre 13 (9-16); post 12 (8-15) | No significant changes |
| Chiara T et al.,  2006 (4) | MEP  FVC  FEV_1_  PEF  Maximal Voluntary Cough | MEP significantly increased following training, *P* = 0.000  PEF (% predicted): Pre 83.56(22.29); post 92.46(21.87); *P* = 0.006  FVC and FEV_1_: no significant differences  Maximal Voluntary Cough: cough volume,rise time,the cough volume acceleration were significant than with the healthy controls | Baseline |
| Chiara T et al.,  2007 (5) | MEP  ALSSS  VRQOL | The difference in MEP from pretraining to posttraining was 26.04 ±16.71 (40.4%)  Posttraining reported significantly less impact of dysarthria  VRQOL: no significant differences | Baseline |
| Fry DK et al.,  2007 (6) | MIP  MEP  FVC  FEV_1_  FEV_1_/FVC  FEF_25-75%_  VC  MVV  FSS | MIP (cmH_2_O): Pre 53.1(25.7); post 76.6(23.3), *P* < 0.001  MIP(% predicted): Pre 53.4(25.4); post 94.0(30.3), *P* < 0.001  MIP (cmH_2_O): Pre 68.7(27.1); post 73.2(22.7), *P* = 0.291  MEP(% predicted): Pre 46.4(19.1); post 49.2(16.6), *P* = 0.355  FVC (L): Pre 3.53(0.75); post 3.73(0.73), *P* = 0.040  FVC(% predicted):Pre 101.4(17.1); post 107.0(13.7), *P* = 0.044  FEV_1_: Pre 2.54(0.63); post 2.77(0.54), *P* = 0.010  FEV_1_(% predicted): Pre 103.1(18.0); post 101.7(17.6), *P* = 0.002  FEV_1_/FVC: Pre 0.724(0.118); post 0.748(0.079), *P* = 0.058  FEV_1_/FVC(% predicted): Pre 0.950(0.149); post 0.990(0.104), *P* = 0.016  FEF_25-75%_: Pre 2.16(0.79); post 2.39(0.87), *P* = 0.020  FEF_25-75%_(% predicted): Pre 72.0(23.1); post 80.3(26.1), *P* = 0.010  VC: Pre 3.64(0.72); post 3.80(0.75), *P* = 0.009  VC(% predicted): Pre 104.2(16.3); post 109.0(14.5), *P* = 0.011  MVV: Pre 93.3(21.4); post 98.3(22.8), *P* = 0.101  MVV(% predicted): Pre 90.5(18.9); post 94.5(20.4), *P* = 0.062  FSS: Pre 5.2(1.1); post 5.2(1.2), *P* = 0.961 | No significant changes |
| Pfalzer L et al.,  2011 (7) | MIP  MEP  MVV  FVC  FEV_1_  FEF_25-75%_  6MWT  GV  FST  SST  Balance testing  FSS  Adherence | MIP (% predicted): Pre 59.3 (29.7); post 94.8 (30.8), increased 71.4%  MEP (% predicted): Pre 45.2 (19.7); post 49.2 (16.6), increased 21.0%  MVV(% predicted): Pre 87.5 (17.9); post 94.5 (20.4), increased 9.0%  FVC: Pre 3.53 (0.75); post 3.73 (0.73)  FVC(% predicted): Pre 101.4 (17.1); post 107.0 (13.7)  FEV_1_: Pre 2.54 (0.63); post 2.77 (0.54)  FEV_1_(% predicted): Pre 103.1 (18.0); post 101.7 (17.6)  FEF_25-75%_: Pre 2.16 (0.79); post 2.39 (0.87)  FEF_25-75%_ (% predicted): Pre 72.0 (23.1); post 80.3 (26.1)  6MWT: Pre 293.9 (170.1); post 306.2 (182.8), *P* =0.086  GV: Pre 0.82 (0.47); post 0.85 (0.51)  FST: Pre 14.2 (10.8); post 14.0 (12.1)  SST: Pre 24.1 (9.8); post 24.1 (13.7), *P* = 0.084  Balance testing,Single limb: Pre 12.6 (6.5); post 19.5 (9.7)  Balance testing,Tandem: Pre 5.6 (3.7); post 8.5 (10.0)  FSS: Pre 47.7 (8.8); post 46.3 (11.3)  Adherence: an average adherence to the IMT training protocol was 81% | No significant changes |
| Ray A D et al.,  2013 (8) | MIP  MEP  FVC  FEV_1_  MVV_12_  6MWT  MFIS  Timed stair climb  MSSE  SF-36  PADS | MIP (cmH_2_O): Pre 70.00(24.00); post 94.00(33.00), *P* < 0.001  MIP (% predicted): Pre 90.00(32.00); post 119.00(39.00)  MEP (cmH_2_O): Pre 94.00(24.00); post 117.00(30.00), *P* < 0.001  MEP (% predicted): Pre 92.00(23.00); post 116.00(31.00)  FVC: Pre 3.39(0.67); post 3.84(0.64), *P* = 0.052  FVC (% predicted): Pre 98.00(18.00); post 101.00(22.00)  FEV_1_: Pre 2.75(0.52); post 2.79(.052), *P* = 0.259  FEV_1_ (% predicted): Pre 99.00(17.00); post 100.00(18.00)  MVV: Pre 95.00(28.00); post 96(23.00), *P* = 0.884  MVV (% predicted): Pre 93.00(23.00); post 94.00(20.00)  6MWT: Pre 428(132); post 431(120), *P* = 0.24  MFIS: Pre 31.0(18.2); post 18.9(12.4), *P* = 0.029  MMSE: Pre 1532.0(251.0); post 1588.0(214.0), *P* = 0.128  SF-36(Phys health): Pre 52.3(44.0); post 84.1(30.2), *P* = 0.031 | MIP (cmH_2_O): Pre 55.00(25.00); post 58.00(21.00), *P* = 0.005  MEP (cmH_2_O): Pre 88.00(26.00); post 85.00(26.00), *P* = 0.002  FVC: Pre 3.62(1.05); post 3.48(1.03), *P* = 0.009  FEV_1_: Pre 3.07(0.95); post 2.79(0.94), *P* = 0.029  MFIS: Pre 20.9(14.2); post 22.4(16.1), *p* = 0.0071 |
| Westerdahl E et al.,  2016 (9) | MIP  MEP  FVC  FEV1  VC  FEV_1_/VC  PEF  EQ-5D VAS | MIP (cmH_2_O): Pre 78(33); post 77(32)  MIP (% predicted): Pre 96(38); post 95(36)  MEP (cmH_2_O): Pre 95(31); post 98(28)  MEP (% predicted): Pre 101(31); post 104(27)  VC: Pre 3.3(0.9); post 3.7(1.0)  FEV_1_: Pre 2.6(0.7); post 2.8(0.7)  FVC: Pre 3.3(0.8); post 3.7(1.1)  FEV_1_/VC: Pre 78.4(7.1); post 74.8(6.6)  PEF: Pre 351(86); post 385(90)  EQ-5D VAS: Pre 64.6(21.5); post 67.3(21.6)  不良反应：discomfort related to the exercises was reported by 4% to a high degree and 13% to some extent. Adverse perceptions were related to dizziness,strenuousness and tediousness. | No significant changes |
| Silverman E P et al.,  2017 (10) | MEP  PAS  SWAL-QOL | MEP (cmH_2_O): Pre 78.60(30.72); post 99.00(32.97), *P* = 0.00042  PAS: 40% demonstrated an improvement in PAS from pre- to post-  treatment; and 15% worsened  SWAL-QOL: Pre 83.3(11.5); post 88.1(10.8), *P* = 0.016 | MEP (cmH_2_O): Pre 75.56(27.68); post 99.38(37.59), *P* = 0.0019  PAS:21.4% worsened, and only 14.3% improved  SWAL-QOL: no significant changes |
| Huang MH et al.,  2020 (11) | MIP  MEP | MIP (cmH_2_O): Pre 25.9(16.4); post 30.6(17.6), *P* = 0.013  MIP (% predicted): Pre 33.2(19.8); post 39.6(22.3), *P* = 0.011  MEP (cmH_2_O): Pre 23.5(15.7); post 24.4(12.9), *P =* 0.639  MEP (% predicted): Pre 25.8(14.4); post 27.4(13.4), *P* = 0.330 | Baseline |
| Martin-Sanchez C et al.,  2020 (12) | MIP  MEP  TV  PEF  MVV  Dyspnea  SF-12  FEV_1_  FEF_25-75_  VC  FVC | MIP (cmH_2_O): Pre 41.83(16.40); post 62.40(24.4), *P* < 0.001  MEP (cmH_2_O): Pre 45.66(20.35); post 62.26(28.44), *P* < 0.001  TV: Pre 0.91(1.14); post 1.33(0.76), *P* < 0.05  PEF: Pre 4.71(2.17); post 5.24(2.37), *P*<0.05  MVV: Pre 60.59(32.46); post 72.57(41.08), *P*<0.001  Dyspnea: Pre 3.58(2.11); post 2.91(2.06), *P*<0.05  SF-12 PCS: Pre 32.91(8.79); post 34.52(8.46), *P*=0.997  SF-12 MCS:Pre 48.56(12.74); post 48.90(9.06), *P* = 0.102  FEV_1_: Pre 2.50(1.06); post 2.53(0.99), *P* = 0.519  FEF_25-75_: Pre 3.27(1.45); post 3.32(1.42), *P* = 0.532  VC: Pre 2.92(1.62); post 3.04(1.58), *P* = 0.799  FVC: Pre 2.70(1.11); post 2.75(1.07), *P* = 0.560  不良反应：only one participant left the program due to fatigue | MIP (cmH_2_O): Pre 48.77(22.68); post 60.54(27.73), *P* < 0.001  MEP (cmH_2_O): Pre 46.64(25.13); post 59.16(30.51), *P* < 0.001  PEF: Pre 4.79(2.00); post 5.38(2.71), *P* < 0.05  MVV: Pre 60.30(38.20); post 79.79(52.13), *P* < 0.001  Dyspnea: Pre 3.48(1.69); post 3.48(2.04), *P* = 0.000  Other outcomes:no significant changes |
| Srp M et al.,  2021 (13) | MEP  vPCF | MEP (cmH_2_O): Pre 87.9(25.3); post 114.3(30.8), *P* = 0.0000  vPCF: Pre 415.9(135.3); post 453.0(146.4), *P* = 0.0036 | Baseline |
| Ghannadi S et al.,  2022 (14) | MIP  MEP  FEV_1_  FVC  FEV_1_/FVC  TLC  RV  TUG  6MWT  MFIS  SF-36 | MIP (cmH_2_O): Pre 44.00(11.21); post 49.75(11.89), *P* < 0.001  MEP (cmH_2_O): Pre 65.71(16.12); post 77.06(19.29), *P* < 0.001  FEV_1_: Pre 3.18(0.74); post 3.10(0.62), *P* < 0.001  FVC: Pre 2.74(0.60); post 3.44(0.73), *P* = 0.001  FEV_1_/FVC(%): Pre 85.65(5.85); post 88.53(4.50), *P* < 0.001  TLC: Pre 4.53(0.85); post 5.10(0.79), *P* < 0.001  RV: Pre 1.69(0.47); post 1.97(0.43), *P* < 0.001  TUG: Pre 13.51(3.39); post 12.39(2.20), *P* = 0.026  6MWT: Pre 417.18(41.25); post 417.529(77.822), *P =* 0.987  MFIS: Pre 28.35(15.88); post 10.71(9.69), *P* < 0.001  SF-36: significantly improved the SF-36 in all subcategories(*p* < 0.001) | MIP (cmH_2_O): Pre 45.58(14.80); post 47.65(16.07), *P* < 0.001  MEP (cmH_2_O): Pre 66.89(15.83); post 69.29(16.34), *P* < 0.001  FEV_1_: Pre 3.17(1.12); post 2.83(0.90), *P* < 0.001  FVC: Pre 2.71(0.83); post 3.30(1.17), *P* < 0.001  FEV_1_/FVC: Pre 86.05(3.42); post 86.09(3.09), *P* = 0.028  RV: Pre 1.79(0.52); post 1.91(0.58), *P* = 0.011  6MWT: Pre 414.21(45.68); post 436.32(63.71), *P* < 0.001  MFIS: Pre 30.41(14.34); post 26.75(13.19), *P* = 0.001  SF-36: physical, social functioning, bodily pain, general health were significantly change.  TLG and TUG: no significant changes |
| Notes:IG, intervention group; CG, control group; NR, not reported; Pre: Outcome measure before intervention. Post: Outcome measure after intervention.  MIP, maximal inspiratory pressure; MEP, maximal expiratory pressure; FVC, forced vital capacity; FEV1, forced expiratory volume in one second; FEV1/FVC, proportion of actual FEV1 to the full FVC; PEF, peak expiratory flow; FEF, forced expiratory flow; VC,vital capacity; TV, tidal volume; TLC: Total Vital Capacity; MVV, Maximal Voluntary Ventilation; PI, pulmonary index; RPE, ; vPCF, Voluntary peak cough flow; 6MWT, 6-Minute Walk test; GV, gait velocity; FST, Functional Stair Test; SST, Sit-to-Stand Test; PADS, Physical Activity Disability Scale; TUG, times up and go test; FSS, Fatigue Severity Scale; MFIS, Modified Fatigue Impact Scale; MSSE, Multiple Sclerosis Self-Efficacy Scale; ALSSS, Amyotrophic Lateral Sclerosis Severity Scale–Speech; EQ-5D VAS, subjective breathing and coughing ability EuroQoL; PAS, penetration and aspiration scale; VRQOL, Voice-Related Quality of Life; SF, Short-Form Health Survey; SWAL-QOL, swallow-related quality of life; RV, residual volume. | | | |

REFERENCES

1. . Smeltzer SC, Lavietes MH, Cook SD. Expiratory training in multiple sclerosis. *Arch Phys Med Rehabil*. (1996) 77: 909-12. doi: 10.1016/s0003-9993(96)90281-6
2. Gosselink R, Kovacs L, Ketelaer P, Carton H, Decramer M. Respiratory muscle weakness and respiratory muscle training in severely disabled multiple sclerosis patients. *Arch Phys Med Rehabil*. (2000) 81: 747-51. doi: 10.1016/s0003-9993(00)90105-9
3. 57. Klefbeck B, Hamrah NJ. Effect of inspiratory muscle training in patients with multiple sclerosis. *Arch Phys Med Rehabil*. (2003) 84: 994-9. doi: 10.1016/s0003-9993(03)00133-3
4. Chiara T, Martin AD, Davenport PW, Bolser DC. Expiratory muscle strength training in persons with multiple sclerosis having mild to moderate disability: effect on maximal expiratory pressure, pulmonary function, and maximal voluntary cough. *Arch Phys Med Rehabil*. (2006) 87: 468-73. doi: 10.1016/j.apmr.2005.12.035
5. Chiara T, Martin D, Sapienza C. Expiratory muscle strength training: speech production outcomes in patients with multiple sclerosis. *Neurorehabil Neural Repair*. (2007) 21: 239-49. doi: 10.1177/1545968306294737
6. Fry DK, Pfalzer LA, Chokshi AR, Wagner MT, Jackson ES. Randomized control trial of effects of a 10-week inspiratory muscle training program on measures of pulmonary function in persons with multiple sclerosis. *J Neurol Phys Ther*. (2007) 31: 162-72. doi: 10.1097/NPT.0b013e31815ce136
7. Pfalzer L, Fry D. Effects of a 10-week inspiratory muscle training program on lower-extremity mobility in people with multiple sclerosis. *International Journal of Ms Care*. (2011) 13: 32-42. doi: 10.7224/1537-2073-13.1.32
8. Ray AD, Udhoji S, Mashtare TL, Fisher NM. A combined inspiratory and expiratory muscle training program improves respiratory muscle strength and fatigue in multiple sclerosis. *Arch Phys Med Rehabil*. (2013) 94: 1964-70. doi: 10.1016/j.apmr.2013.05.005
9. Silverman EP, Miller S, Zhang Y, Hoffman-Ruddy B, Yeager J, Daly JJ. Effects of expiratory muscle strength training on maximal respiratory pressure and swallow-related quality of life in individuals with multiple sclerosis. *Multiple Sclerosis Journal - Experimental, Translational and Clinical*. (2017) 3: 1842836499. doi: 10.1177/2055217317710829
10. Westerdahl E, Wittrin A, Kånåhols M, Gunnarsson M, Nilsagård Y. Deep breathing exercises with positive expiratory pressure in patients with multiple sclerosis - a randomized controlled trial. *Clin Respir J*. (2016) 10: 698-706. doi: 10.1111/crj.12272
11. Huang MH, Fry D, Doyle L, Burnham A, Houston N, Shea K, et al. Effects of inspiratory muscle training in advanced multiple sclerosis. *Mult Scler Relat Disord*. (2020) 37: 101492. doi: 10.1016/j.msard.2019.101492
12. Martin-Sanchez C, Calvo-Arenillas JI, Barbero-Iglesias FJ, Fonseca E, Sanchez-Santos JM, Martin-Nogueras AM. Effects of 12-week inspiratory muscle training with low resistance in patients with multiple sclerosis: a non-randomised, double-blind, controlled trial. *Mult Scler Relat Disord*. (2020) 46: 102574. doi: 10.1016/j.msard.2020.102574
13. Srp M, Capek V, Gal O, Havrdova EK, Jech R, Korteova R, et al. Severely disabled multiple sclerosis patients can achieve the performance of healthy subjects after expiratory muscle strength training. *Mult Scler Relat Dis*. (2021) 55: 103187. doi: 10.1016/j.msard.2021.103187
14. Ghannadi S, Noormohammadpour P, Mazaheri R, Sahraian MA, Mansournia MA, Pourgharib SM, et al. Effect of eight weeks respiratory muscle training on respiratory capacity, functional capacity and quality of life on subjects with mild to moderate relapsing-remitting multiple sclerosis: a single-blinded randomized controlled trial. *Mult Scler Relat Disord*. (2022) 68: 104208. doi: 10.1016/j.msard.2022.104208
