# Supplementary material for: Effectiveness of respiratory muscle training in adults with multiple sclerosis: a systematic review and meta-analysis
Source: Front Neurol. 2025 Oct 23;16:1665651. doi: 10.3389/fneur.2025.1665651 (PMC12590560; doi:10.3389/fneur.2025.1665651)
Supplement: Supplementary file 3 [file Table_3.doc]

| **Effect of** **respiratory muscle training in adults with multiple sclerosis: A systematic review and meta-analysis**  **Supplement figure 1 . Subgroup analyses of primary outcome** |
| --- |
| **A:MIP (cmH_2_O)** |
| **（1）Type of Studies** |
| **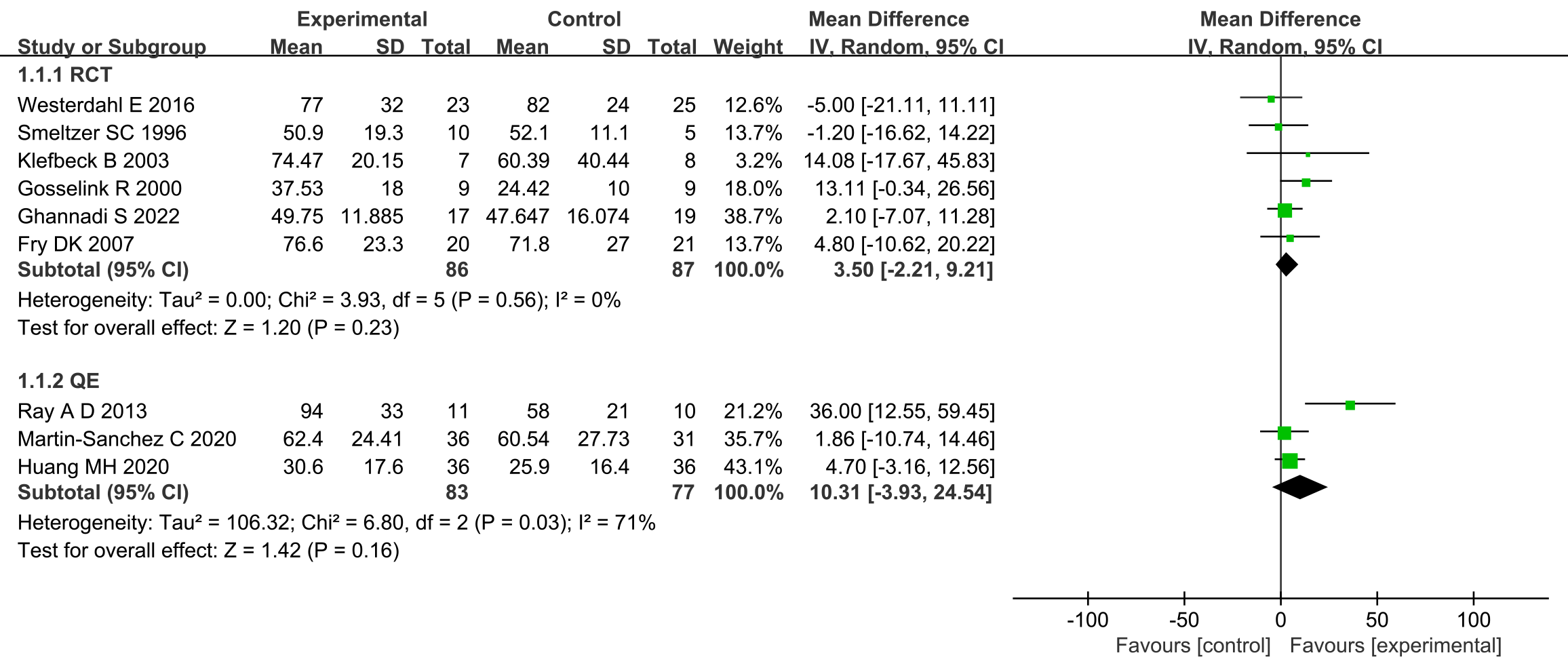** |
| **（2）Type of Interventions** |
| **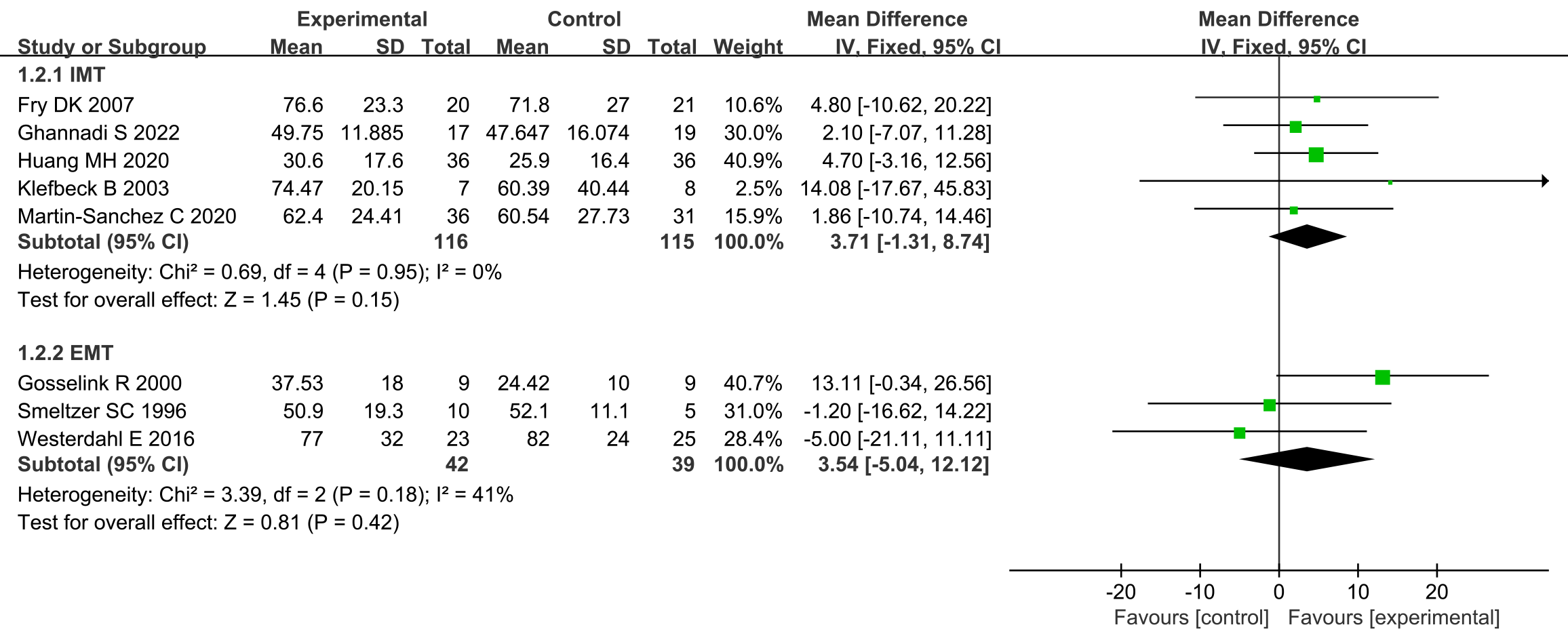** |
| **（3）Disability level** |
| **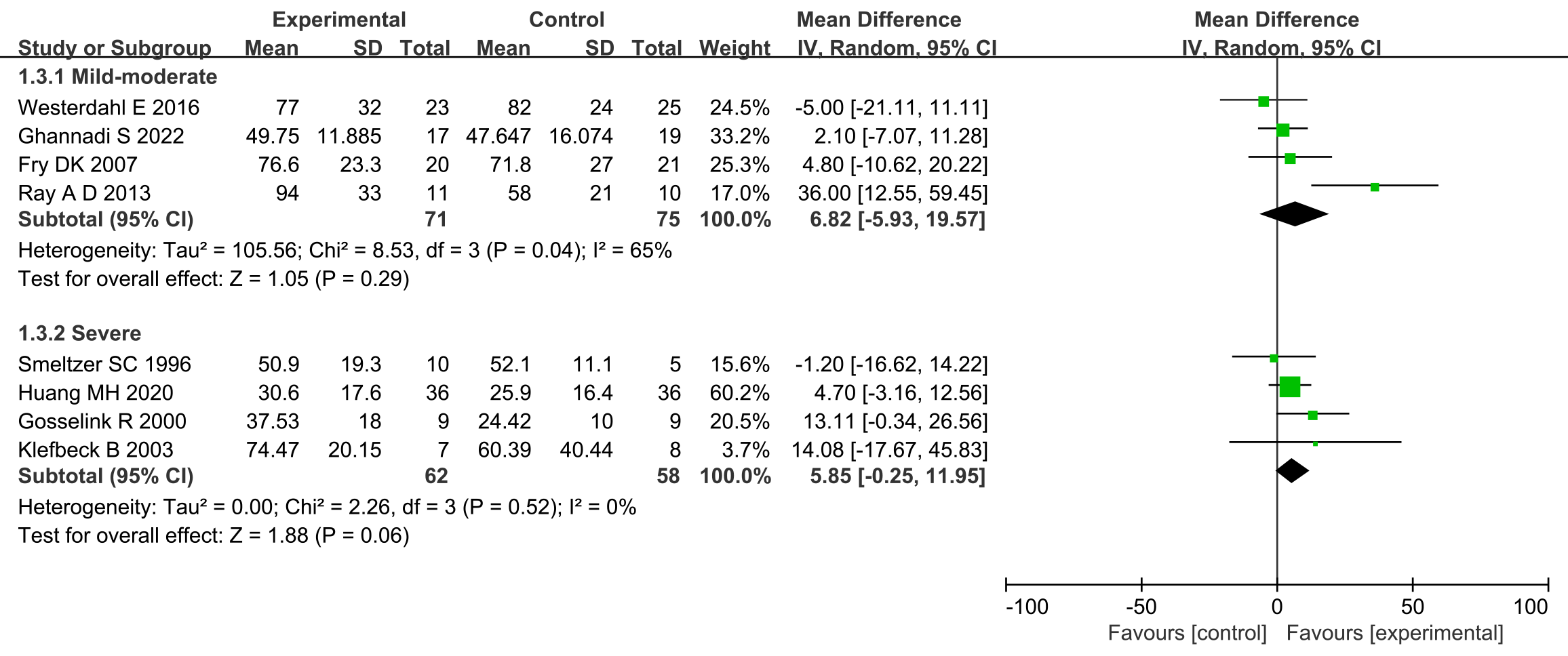** |
| **（4）Intervention duration** |
| 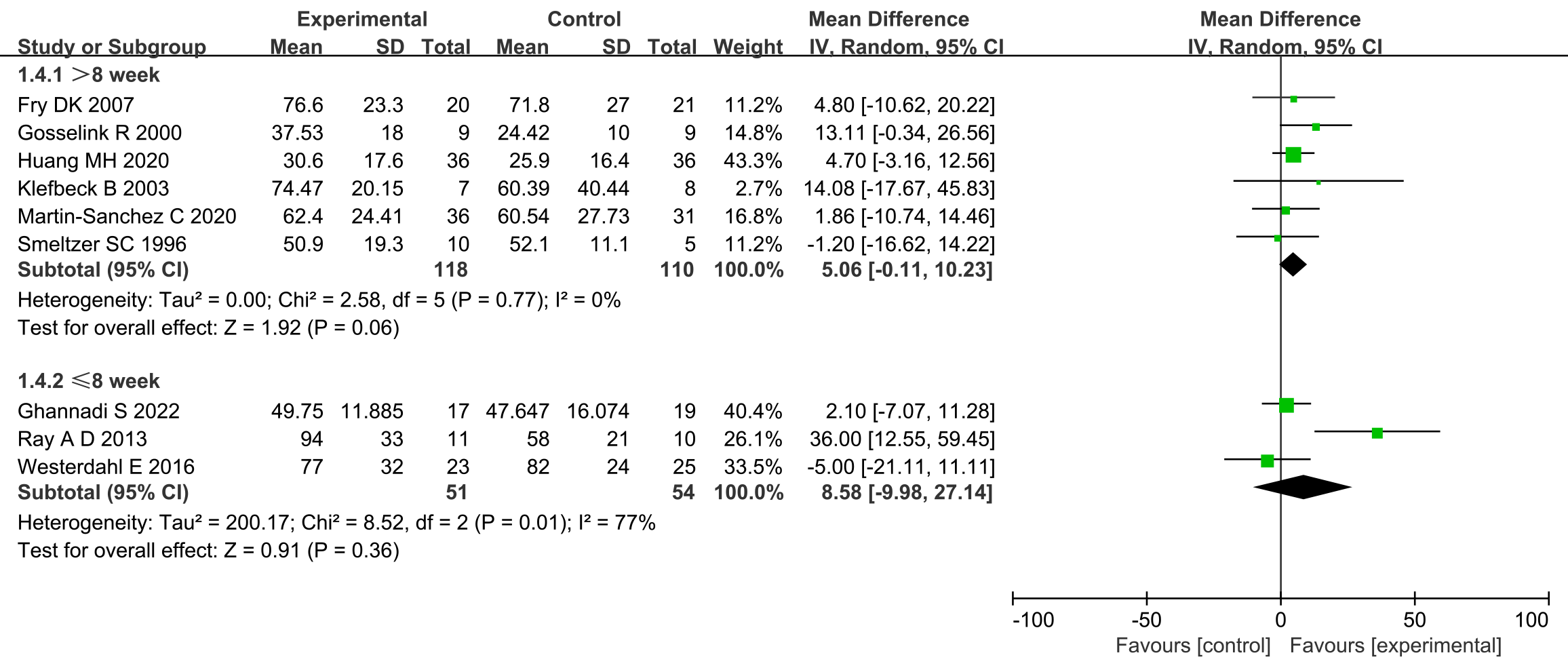 |

| **B:MIP (% predicted)** |
| --- |
| **（1）Type of Studies** |
| **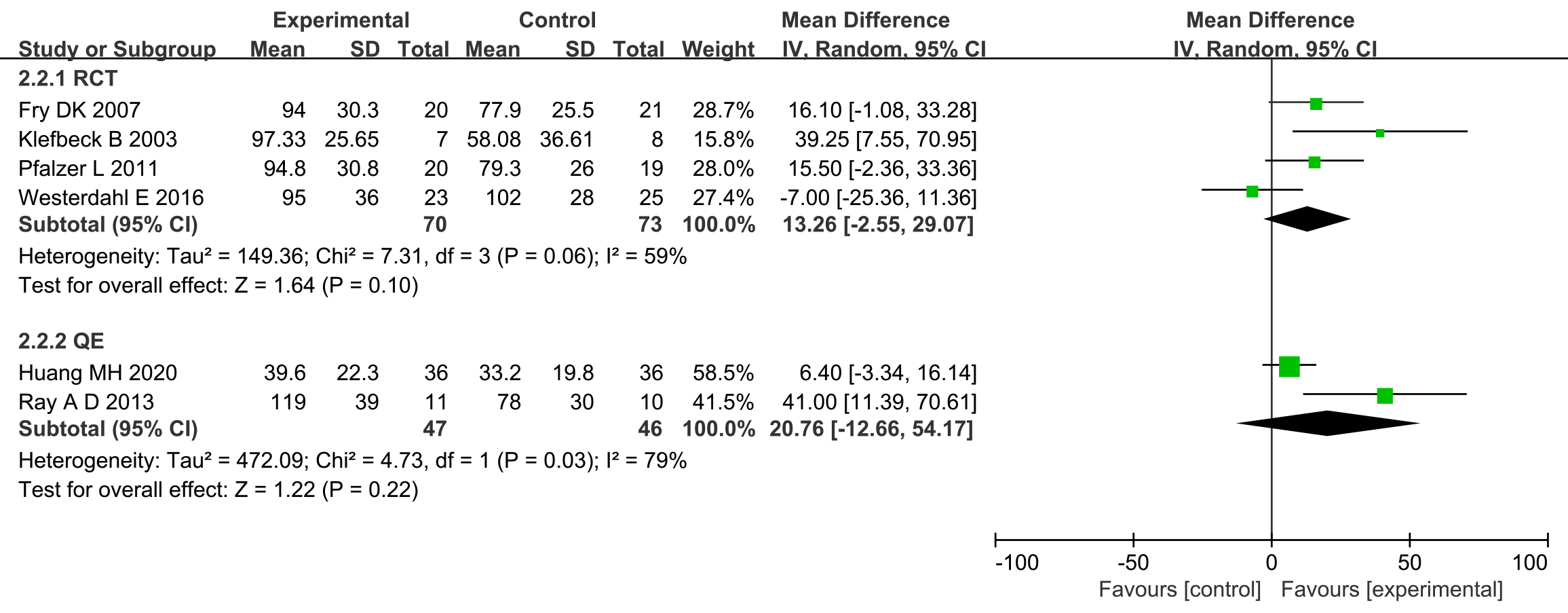** |
| **（2）Type of Interventions** |
| **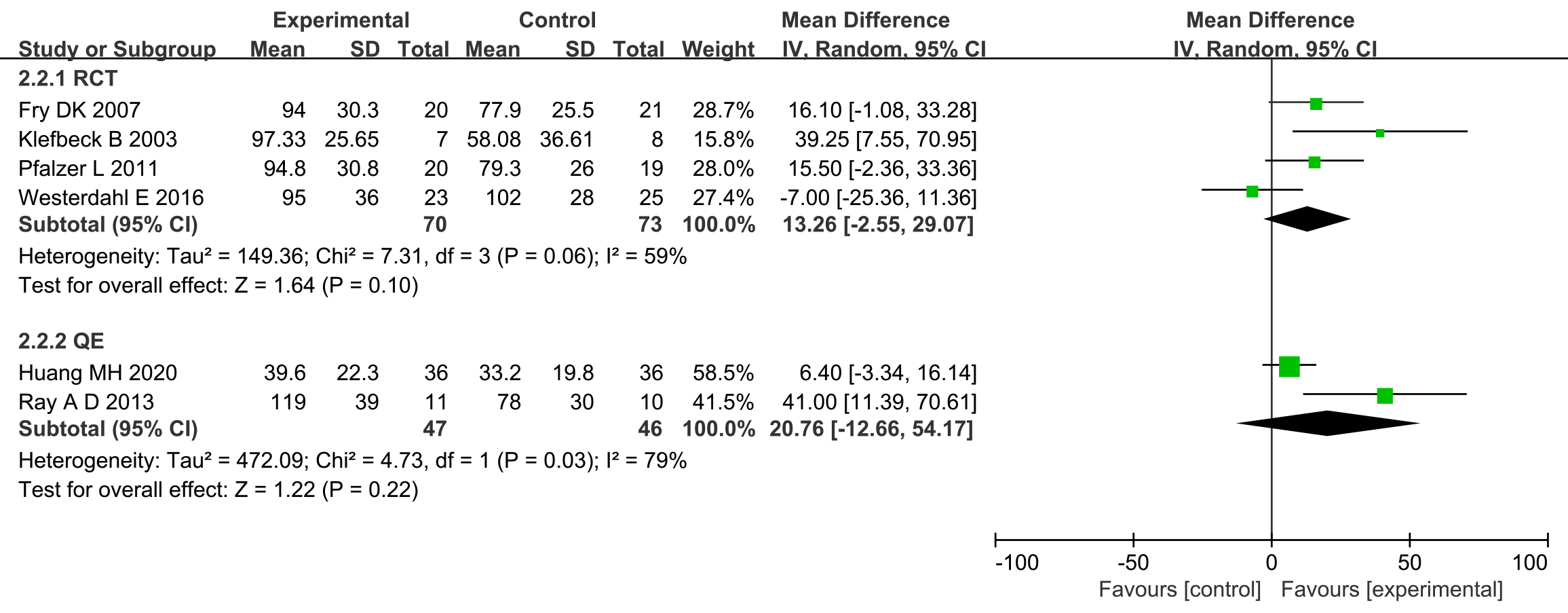** |
| **（3）Disability level** |
| **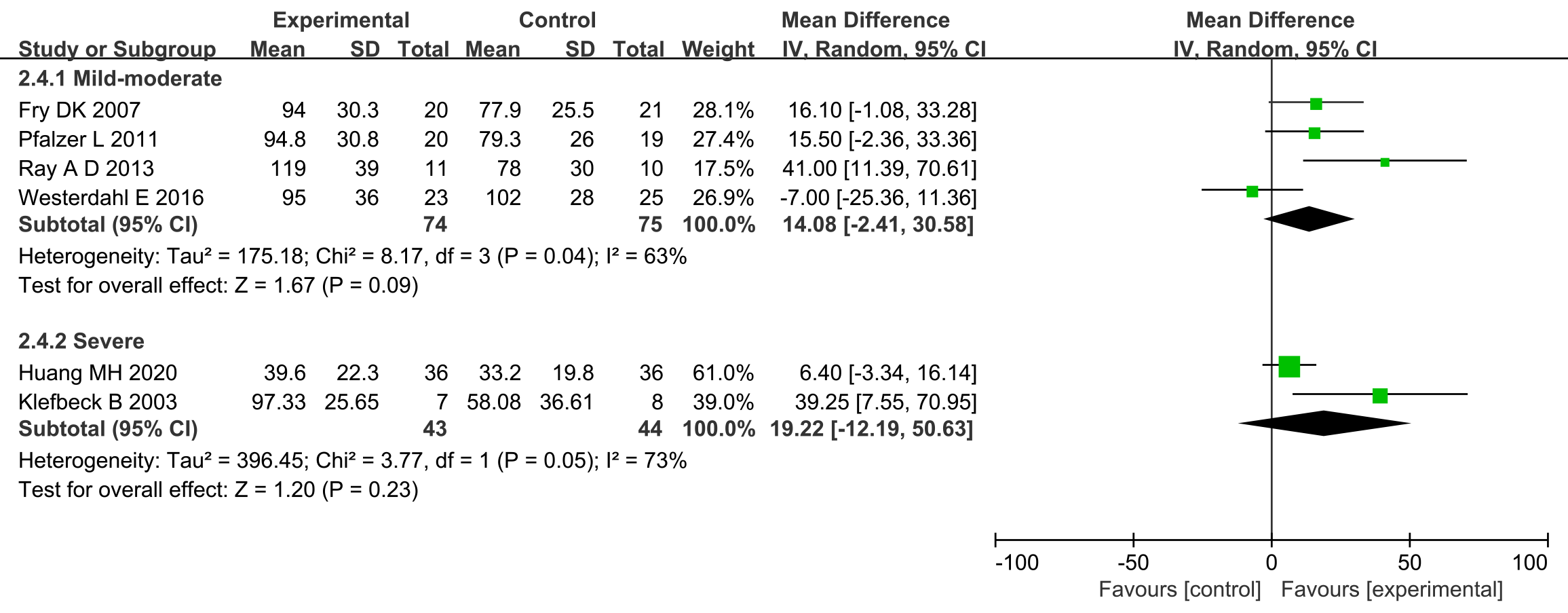** |
| **（4）IIntervention duration** |
| 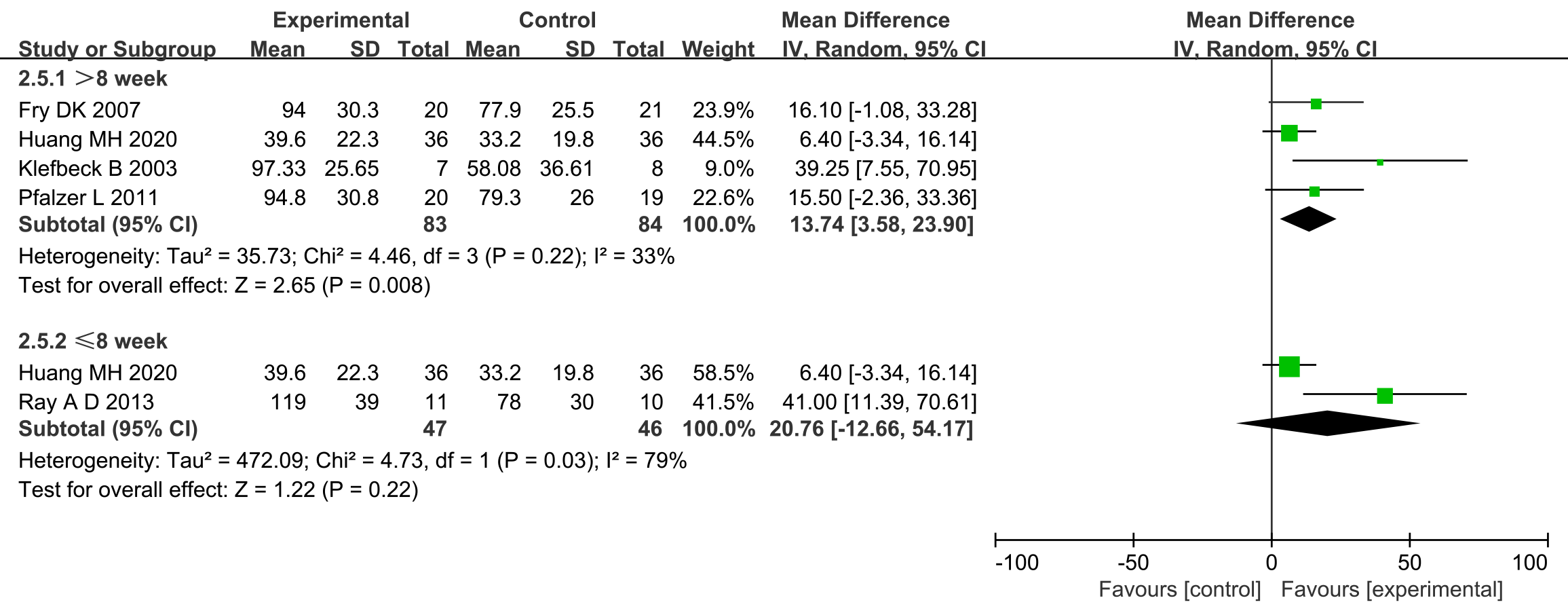 |

| **C:MEP (cmH_2_O)** |
| --- |
| **（1）Type of Studies** |
| **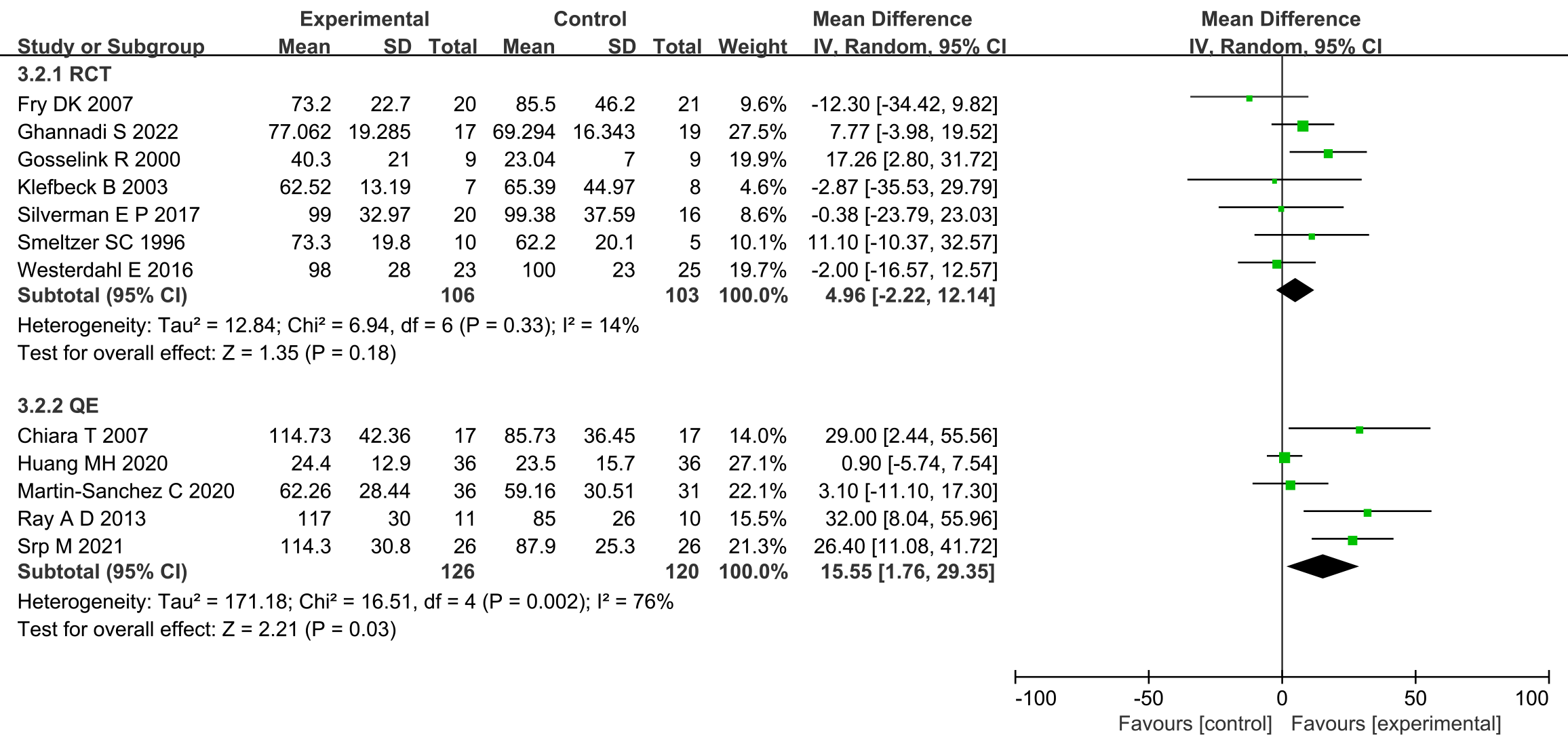** |
| **（2）Type of Interventions** |
| **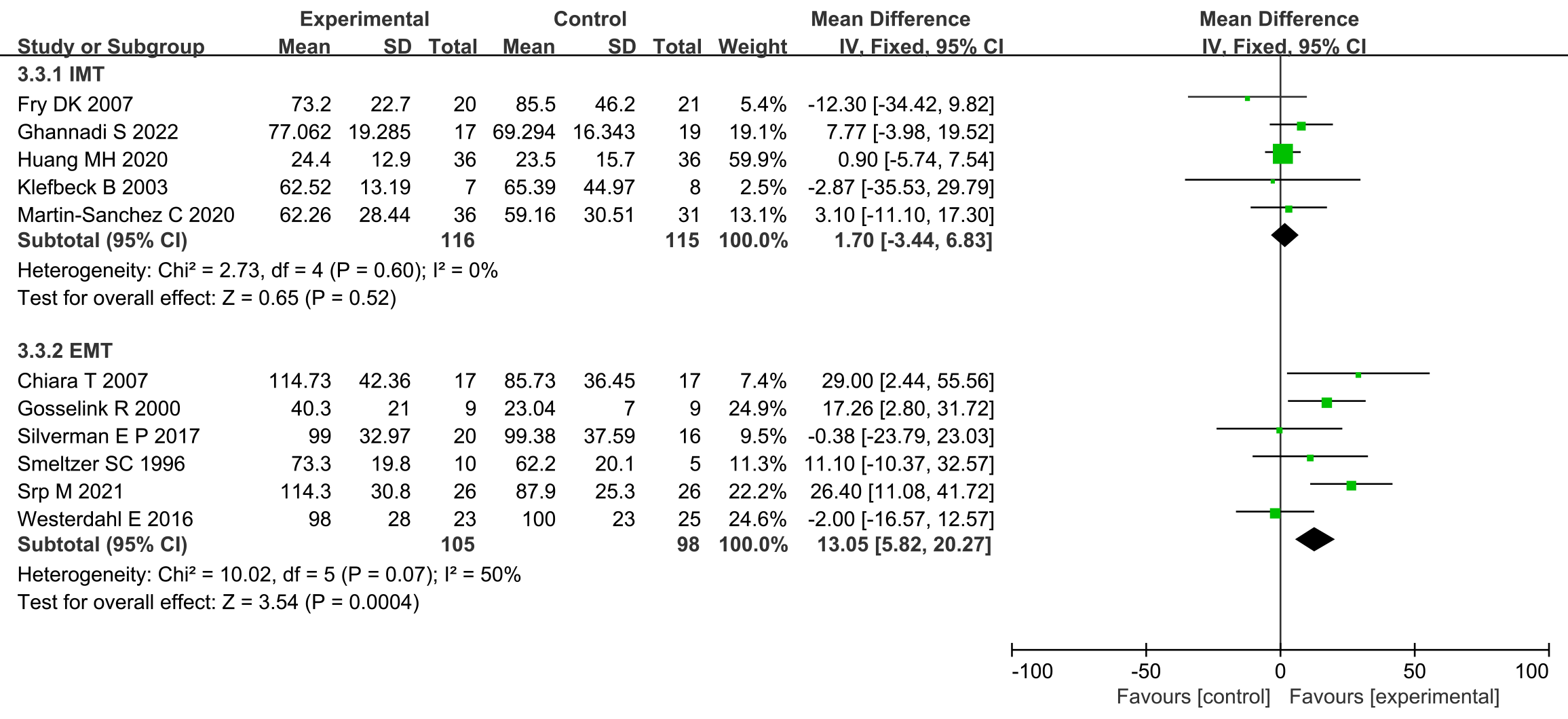** |
| **（3）Disability level** |
| **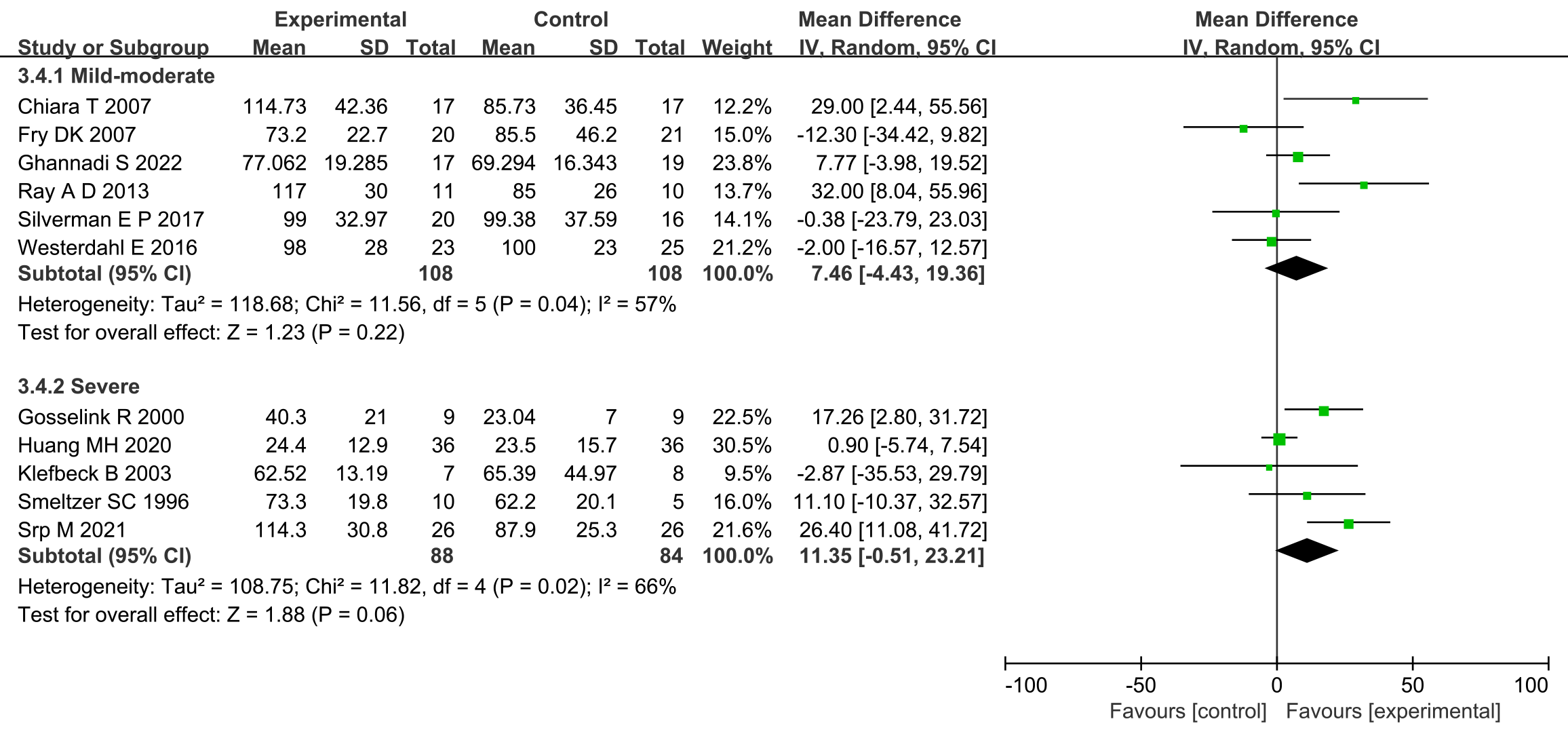** |
| **（4）Intervention duration** |
| 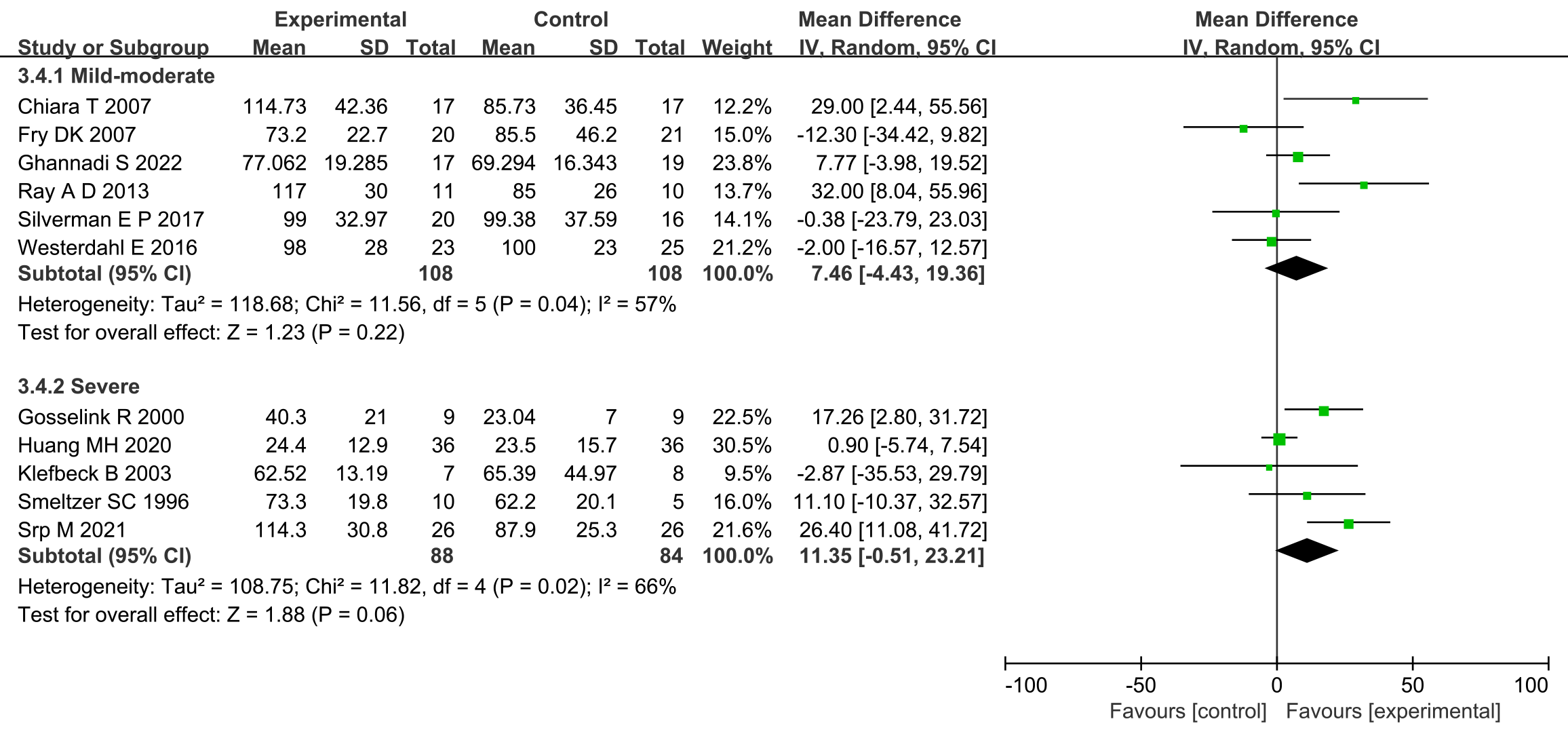 |

| **D:MEP (% predicted)** |
| --- |
| **（1）Type of Studies** |
| **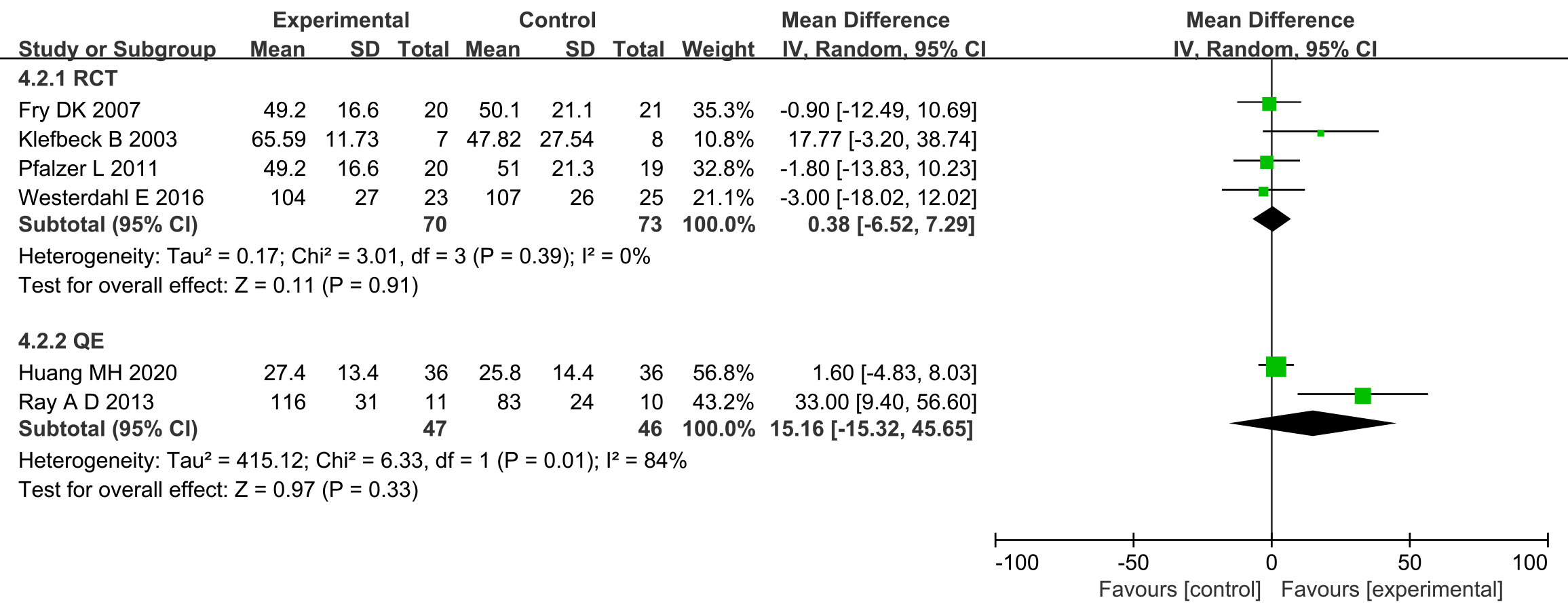** |
| **（2）Type of Interventions** |
| **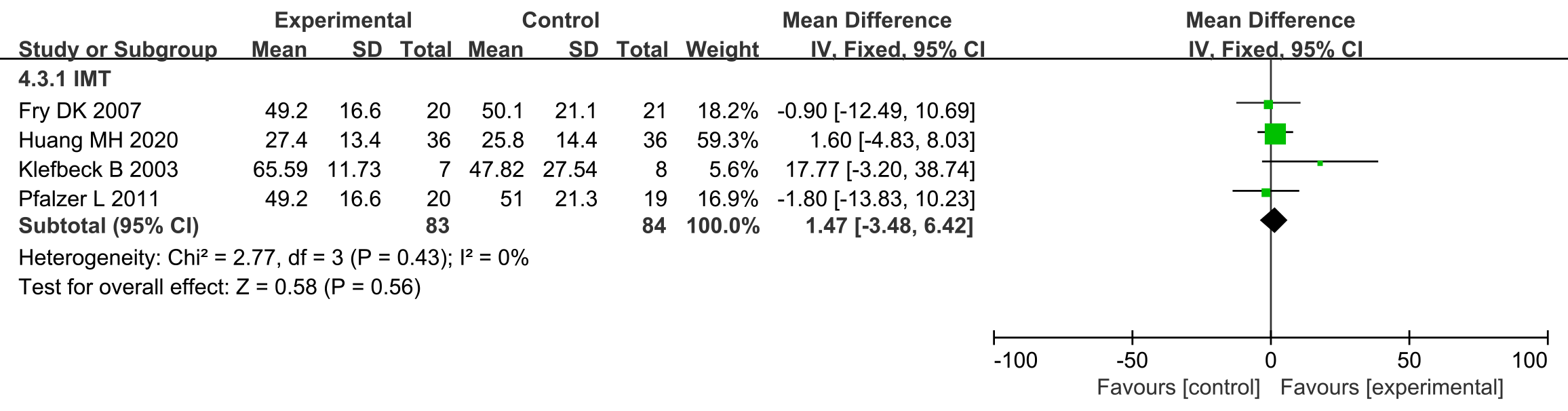** |
| **（3）Disability level** |
| **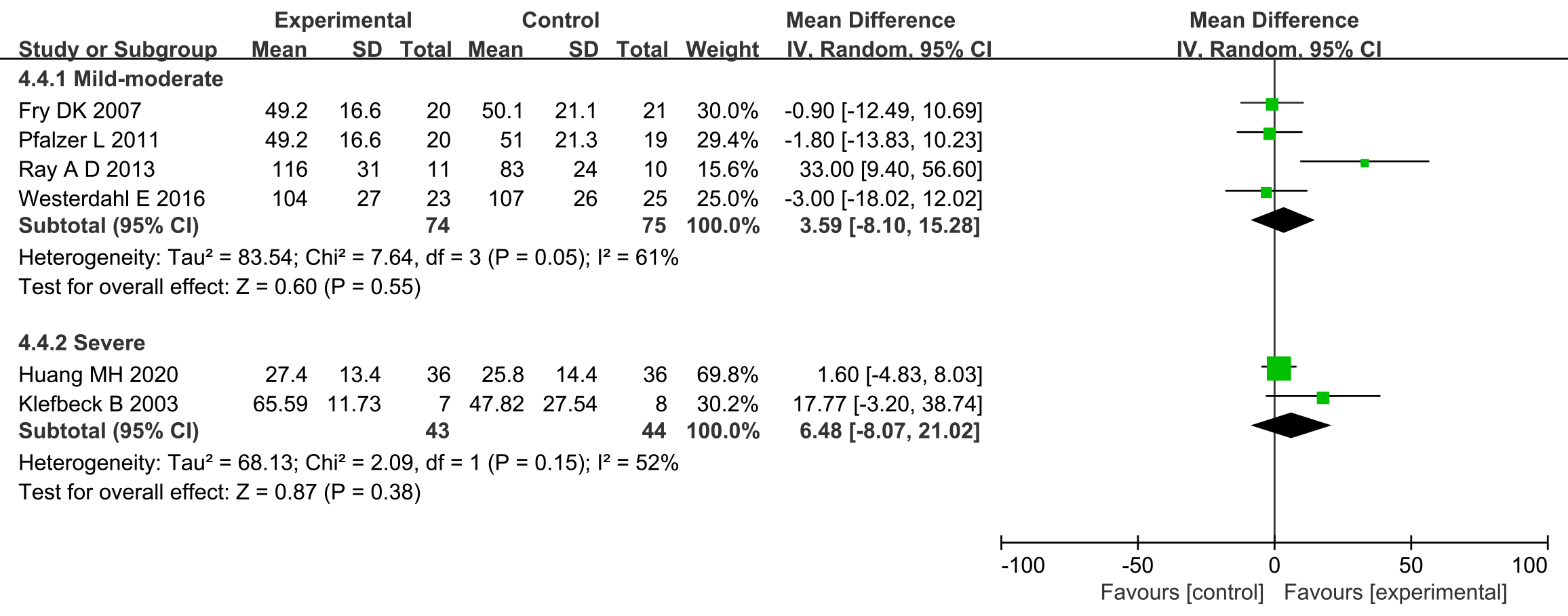** |
| **（4）Intervention duration** |
| 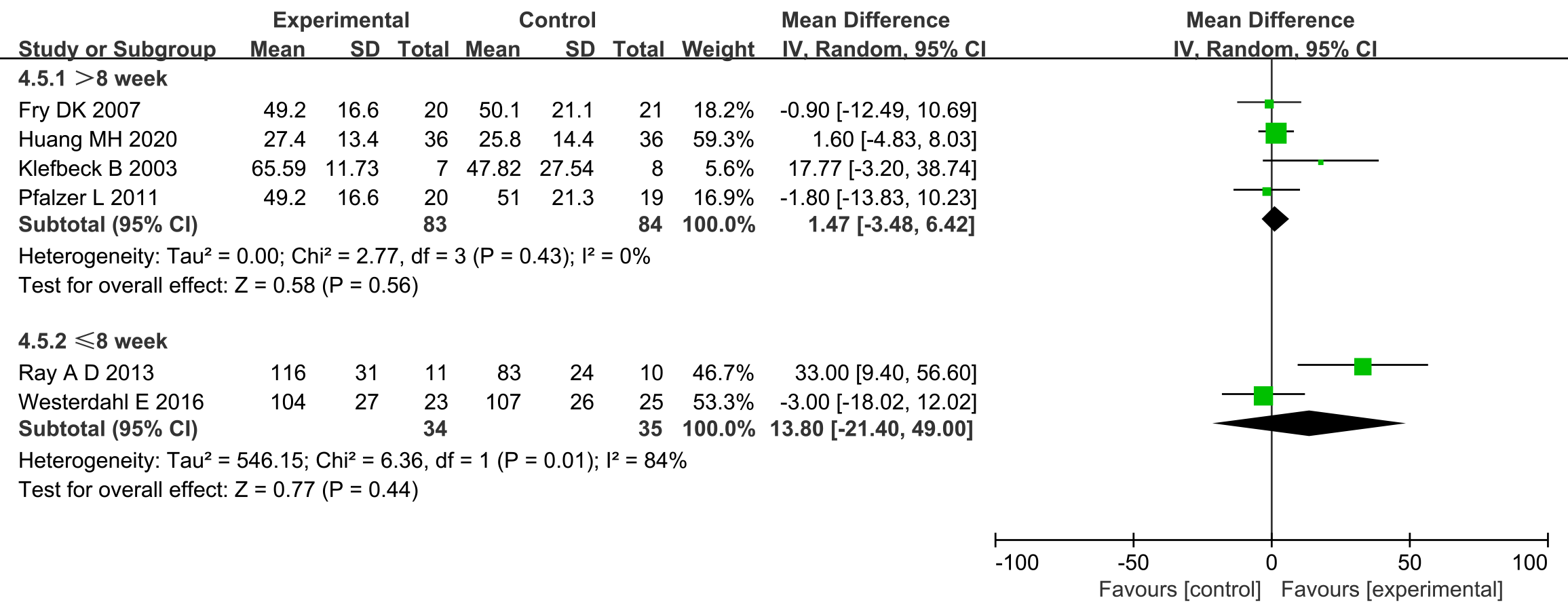 |
