# Supplementary material for: Effectiveness of respiratory muscle training in adults with multiple sclerosis: a systematic review and meta-analysis
Source: Front Neurol. 2025 Oct 23;16:1665651. doi: 10.3389/fneur.2025.1665651 (PMC12590560; doi:10.3389/fneur.2025.1665651)
Supplement: Supplementary file 4 [file Table_4.docx]

**Effect of** **respiratory muscle training in adults with multiple sclerosis: A systematic review and meta-analysis**

| **Supplement table 3. The agreements and disagreement with published systematic review and meta-analysis** | | | | | | | |
| --- | --- | --- | --- | --- | --- | --- | --- |
| **First author, Publication year** | **Criteria for considering studies for this review** | | | | | **Search**  **Deadline** | **Results** |
|  | **Studies** | **Participants** | **Intervention** | **Control** | **Outcomes** |  |  |
| Martín-Valero R etal.,  2014 | RCT  Non-RCT  QE  Cohort  Case study  SR  Cohort | MS | IMT and EMT  through threshold | Not receive the therapeutic intervention | ①Respiratory muscle strength (MIP,MEP)  ②Physical capacity (6MWT)  ③Clinical outcomes and others. | 2013 | Included 15 trials, 4 trails received EMT (2 RCTs, 1 non-RCT), 3 trails of IMT (3 RCTs), 1 trail combined IMT and EMT (QE), 7 trails showed the relationship between the degree of disability and the impairment at both the physical and pulmonary function (2 SR, 3 study cases, 1 N-RCT, 1 RCT).  ①Five RCTs (3 IMT[1-3], 2 EMT[4,5]) pooled by meta-analysis: effect size of MIP (MD 10.01 cmH_2_O, 95%CI 4.29-15.74, *P*＜0.01) and MEP (MD cmH_2_O 6.27, 95%CI 1.18-11.36, *P*＜0.001).  ②Significant changes were found in FVC (*P*＜0.001), pulmonary dysfunction index (*P*＜0.05), and quality of life in comparison with control group. |
| Campbell E et al., 2016 | RCT | MS | Physiotherapy Rehabilitation | NR | NR | 2014 | Included 1 trial[1]，a significant improvement was found in MIP and MEP in those using IMT. No changes were seen in any other outcomes |
| Ferreira G D et al., 2016 | RCT | MS or ALS | RMT | Not received RMT or received training without load | ①Respiratory muscle strength (MIP,MEP)  ②Pulmonary function (FVC,FEV_1_,MVV）  ③Physical capacity (6MWT) | January 2015 | Included 6 trials were performed with MS patients.  ①Respiratory muscle strength:  MIP[1-5,9] (MD cmH_2_O 22.5, 95%CI 3.4-41.5), (*I*^2^=90%, Random) ;  MEP[1-5,9] (MD cmH_2_O 8.4, 95%CI 3.6-13.2), (*I*^2^=44%, Random) ;  ②Pulmonary function:  FVC [2,3,5,9] (MD 0.40 L, 95%CI -0.27, 1.07), (*I*^2^=0%, Random);  FEV_1_ [2,3,9] (MD 0.27 L, 95%CI 0.12, 0.42), (*I*^2^=0%, Random);  MVV [2,3,9] (MD 0.88 L/min, 95%CI -5.47, 7.24), (*I*^2^=92%, Random);  ③Physical capacity:  6MWD [3,9] (MD 18 m, 95%CI -4.54, 40.44), (*I*^2^=70%, Random) |

| **Continue Supplement 4. The agreements and disagreement with published systematic review and meta-analysis** | | | | | | | | | | |
| --- | --- | --- | --- | --- | --- | --- | --- | --- | --- | --- |
| **First author, Publication year** | **Criteria for considering studies for this review** | | | | | **Search**  **Deadline** | **Results** | | | |
|  | **Studies** | **Participants** | **Intervention** | **Control** | **Outcomes** |  |  |  |  |  |
| Rietberg MB et al., 2017 | RCT | Adults of MS | RMT (strength and endurance of inspiratory and expiratory muscles) | general rehabilitation or physical therapy  exercise training  functional training  home physical training  aquatic therapy  technology and equipment in daily living  Electrotherapy  electrical muscle  nerve stimulation  no training | ①Respiratory muscle strength (MIP, MEP)  ②Quality of life  ③Pulmonary function (forced expiratory volume, FVC and peak flow)  ④Clinical pulmonary parameters  ⑤Fatigue (FSS)  ⑥Adverse events | February 2017 | Included 6 trials were performed with 195 MS patients. | | | |
|  |  |  |  |  |  |  | Indicators | No. of studies | Sample | Effect size  (MD, 95%CI) |
|  |  |  |  |  |  |  | 1 IMT [1,2] | | | |
|  |  |  |  |  |  |  | 1.1 MIPcmH_2_O | 2 | 56 | (6.50, 95%CI -7.39, 20.38, *P*=0.36) |
|  |  |  |  |  |  |  | 1.2 MIP(% Prediction) | 2 | 56 | (20.92, 95%CI 6.03, 35.81, *P*=0.006) |
|  |  |  |  |  |  |  | 1.3 MEPcmH_2_O | 2 | 56 | (-8.22, 95%CI -26.20, 9.77, *P*=0.37) |
|  |  |  |  |  |  |  | 1.4 MEP(% Prediction) | 2 | 56 | (5.86, 95%CI -10.63, 22.35, *P*=0.49) |
|  |  |  |  |  |  |  | 1.5 FSS | 2 | 56 | (-0.28, 95%CI -0.95, 0.39, *P*=0.42) |
|  |  |  |  |  |  |  | 2 EMT[4,5,7] | | | |
|  |  |  |  |  |  |  | 2.1 MIPcmH_2_O | 3 | 81 | (3.54, 95%CI -5.04, 12.12, *P*=0.42) |
|  |  |  |  |  |  |  | 2.2 MEPcmH_2_O | 3 | 81 | (8.33, 95%CI -0.93, 17.59, *P*=0.18) |
|  |  |  |  |  |  |  | ②Qualitative analysis was performed on the other outcomes | | | |
| Levy J et al.,  2018 | Clinical trials and cohorts | MS | Respiratory rehabilitation | NR | NR | December 2016 | Eleven studies were retained for review. Seven RCTs [1-7], 2 non-RCTs[8,9], and 2 observational studies. RMT (inspiratory and/or expiratory) by use of a portable resistive mouthpiece was the most frequently evaluated technique. All reviewed studies evaluated home-based rehabilitation programs and focused on spirometric outcomes. The disparities in outcome measures among published studies did not allow for a meta-analysis and cough assistance devices were not evaluated in this population. | | | |
| Notes: RMT,respiratory muscle training; IMT,Inspiratory muscle training; EMT,expiratory muscle training; RCT, randomized controlled trials; QE, quasi-experimental trials; SR, systematic reviews; MIP, maximal inspiratory pressure; MEP, maximal expiratory pressure; ALS, Amyotrophic lateral sclerosis; FVC, Forced vital capacity; FEV1, Forced expiratory volume in one second; MVV, Maximum voluntary ventilation | | | | | | | | | | |

**Reference**

[1]Klefbeck B, Hamrah NJ. Effect of inspiratory muscle training in patients with multiple sclerosis. Arch Phys Med Rehabil 2003;84(7):994-9. <https://doi.org/10.1016/s0003-9993(03)00133-3.>

[2]Fry DK, Pfalzer LA, Chokshi AR, Wagner MT, Jackson ES. Randomized control trial of effects of a 10-week inspiratory muscle training program on measures of pulmonary function in persons with multiple sclerosis. J Neurol Phys Ther 2007;31(4):162-72.

[3] Pfalzer L, Fry D. Effects of a 10-week inspiratory muscle training program on lower-extremity mobility in people with multiple sclerosis. International Journal of Ms Care 2011;13(1):32-42. <https://doi.org/10.7224/1537-2073-13.1.32.>

[4] Smeltzer SC, Lavietes MH, Cook SD. Expiratory training in multiple sclerosis. Arch Phys Med Rehabil 1996;77(9):909-12. <https://doi.org/10.1016/s0003-9993(96)90281-6.>

[5] Gosselink R, Kovacs L, Ketelaer P, Carton H, Decramer M. Respiratory muscle weakness and respiratory muscle training in severely disabled multiple sclerosis patients. Arch Phys Med Rehabil 2000;81(6):747-51. <https://doi.org/10.1016/s0003-9993(00)90105-9.>

[6] Mutluay FK, Demir R, Ozyilmaz S, Caglar AT, Altintas A, Gurses HN. Breathing-enhanced upper extremity exercises for patients with multiple sclerosis. Clin Rehabil 2007;21(7):595-602. <https://doi.org/10.1177/0269215507075492.>

[7] Westerdahl E, Wittrin A, Kånåhols M, Gunnarsson M, Nilsagård Y. Deep breathing exercises with positive expiratory pressure in patients with multiple sclerosis -a randomized controlled trial. Clin Respir J 2016;10(6):698-706. <https://doi.org/10.1111/crj.12272.>

[8] Chiara T, Martin AD, Davenport PW, Bolser DC. Expiratory muscle strength training in persons with multiple sclerosis having mild to moderate disability: effect on maximal expiratory pressure, pulmonary function, and maximal voluntary cough. Arch Phys Med Rehabil 2006;87(4):468-73. <https://doi.org/10.1016/j.apmr.2005.12.035.>

[9] Ray AD, Udhoji S, Mashtare TL, Fisher NM. A combined inspiratory and expiratory muscle training program improves respiratory muscle strength and fatigue in multiple sclerosis. Arch Phys Med Rehabil 2013;94(10):1964-70. <https://doi.org/10.1016/j.apmr.2013.05.005.>
